# A Portable Soft Robotic Glove with Fully Functional Thumb Assistance for Complex Dexterous Fine Motor Skills

**DOI:** 10.1002/advs.76275

**Published:** 2026-06-26

**Authors:** Disheng Xie, Zhen Wang, Zhongping Ye, Minghao Liu, Xiangqian Shi, Shuk Fan Tong, Jianbin Liu, Chuanbin Mao, Thomas W. Leung, Haitao Liu, Raymond Kai‐yu Tong

**Affiliations:** ^1^ Department of Biomedical Engineering the Chinese University of Hong Kong Hong Kong SAR China; ^2^ Key Laboratory of Mechanism Theory and Equipment Design of Ministry of Education Tianjin University Tianjin China; ^3^ Faculty of Medicine the Chinese University of Hong Kong Hong Kong SAR China

**Keywords:** assist as need, origami actuator, rehabilitation, soft robotic glove, thumb

## Abstract

Stroke causes millions of cases of hand disability annually, impairing fine motor skills needed for daily tasks like pinching, circumduction, phone swiping, cap twisting, and thumb opposition. Existing hand exoskeletons and soft gloves often lack adequate thumb support, are bulky, and rely on unsensorized open‐loop control. We developed a portable soft robotic glove that provides comprehensive, task‐oriented assistance for the thumb and fingers. A novel soft continuous‐segmented dual‐chamber origami thumb actuator enables active carpometacarpal abduction/adduction, flexion/extension, metacarpophalangeal flexion/extension, and interphalangeal extension, covering the full functional range and generating 18 N tip force–about twice what's required to overcome typical post‐stroke spasticity. The glove integrates a sensorized portable driving and an “assist‐as‐needed”controller to minimize support and maximize user engagement. Three chronic stroke participants with varied impairment levels performed pinching, lateral pinching, circumduction, phone swiping, cap twisting, and thumb opposition while wearing the glove; all completed the tasks. Across participants, the glove increased lateral pinch force by 62.9% and expanded thumb range of motion ninefold. These results indicate the system's potential as a practical solution for dexterous, task‐oriented rehabilitation to restore functional hand movements and improve daily living.

## Graphical abstract

A portable, sensorized, and controlled dexterous thumb‐enhanced glove helps stroke survivors swipe phones, twist caps, and perform other tasks. It is based on a powerful continuously, segmented origami dual chamber actuator to achieve active thumb CMC and MCP joint assistance and passively extend the IP joint.

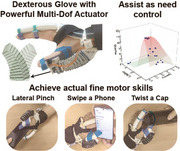

## 1 Introduction

Every year, strokes lead to millions of disabilities [1], with over half of patients facing difficulties in hand functions that are essential for activities of daily living (ADLs) [2]. Physical therapy [3] is the primary intervention that helps patients regain hope for limb control. However, the labor‐intensive nature of this therapy, combined with a shortage of qualified physical therapists [4] presents significant challenges. In response, robotic‐assisted rehabilitation [5] has emerged over the past few decades, with innovations such as hand exoskeletons [6] and soft robotic gloves [7] providing repetitive and task‐oriented training [8, 9], aiming to address existing shortcomings. These years, evidence indicates that task‐oriented training, particularly when delivered with varying degrees of complexity that mimic daily activities, enhances neuroplasticity and functional outcomes [10]. Specifically, the diversity of tasks addressed by assistive devices correlates positively with better recovery. For hand exoskeletons or soft robotic gloves, having greater degrees of freedom to facilitate finger and thumb movements across a wider range of daily activities leads to improved rehabilitation outcomes. Therefore, the following section reviews the advantages and limitations of various robotic‐assisted rehabilitation designs, focusing on this critical aspect. Note S1 and Table S1 also provide a detailed comparison of existing methods from more aspects such as weight, response time, etc.

Hand exoskeletons first appeared with designs that use motors to drive finger movements through rigid links [11, 12] showing promise in facilitating recovery through repetitive hand opening and closing movement training [13, 14]. They are capable of driving the fingers and thumb to flex and extend. Although thumb improved design has emerged with greater degrees of freedom (DOFs) [15, 16, 17], their large volume causes problem to be integrated into daily life [4] and heavy weight and stiffness impose a burden on stroke survivors with weakened upper limbs [18, 19], limiting their use in task‐oriented training [20]. To solve aforementioned drawbacks, researchers tend to remove the rigid links away and drive the finger using actuators through lightweight cables forming the cable‐driven soft robotics gloves [21, 22, 23, 24], achieving assistance around carpometacarpal (CMC) joint [25, 26]. The routing of pulling cables between the palm and thenar eminence remains difficult, limiting thumb adduction assistance. Soft robotic gloves driven by soft actuators [27, 28] have emerged with inherent compliance for natural movement and self‐alignment properties. These gloves [29, 30] demonstrate improved rehabilitative effects [31] compared to hand exoskeletons. However, most soft robotic gloves primarily assist the fingers and thumb in flexion and extension, making ADLs such as twisting caps and swiping phones impossible.

**FIGURE 1:**
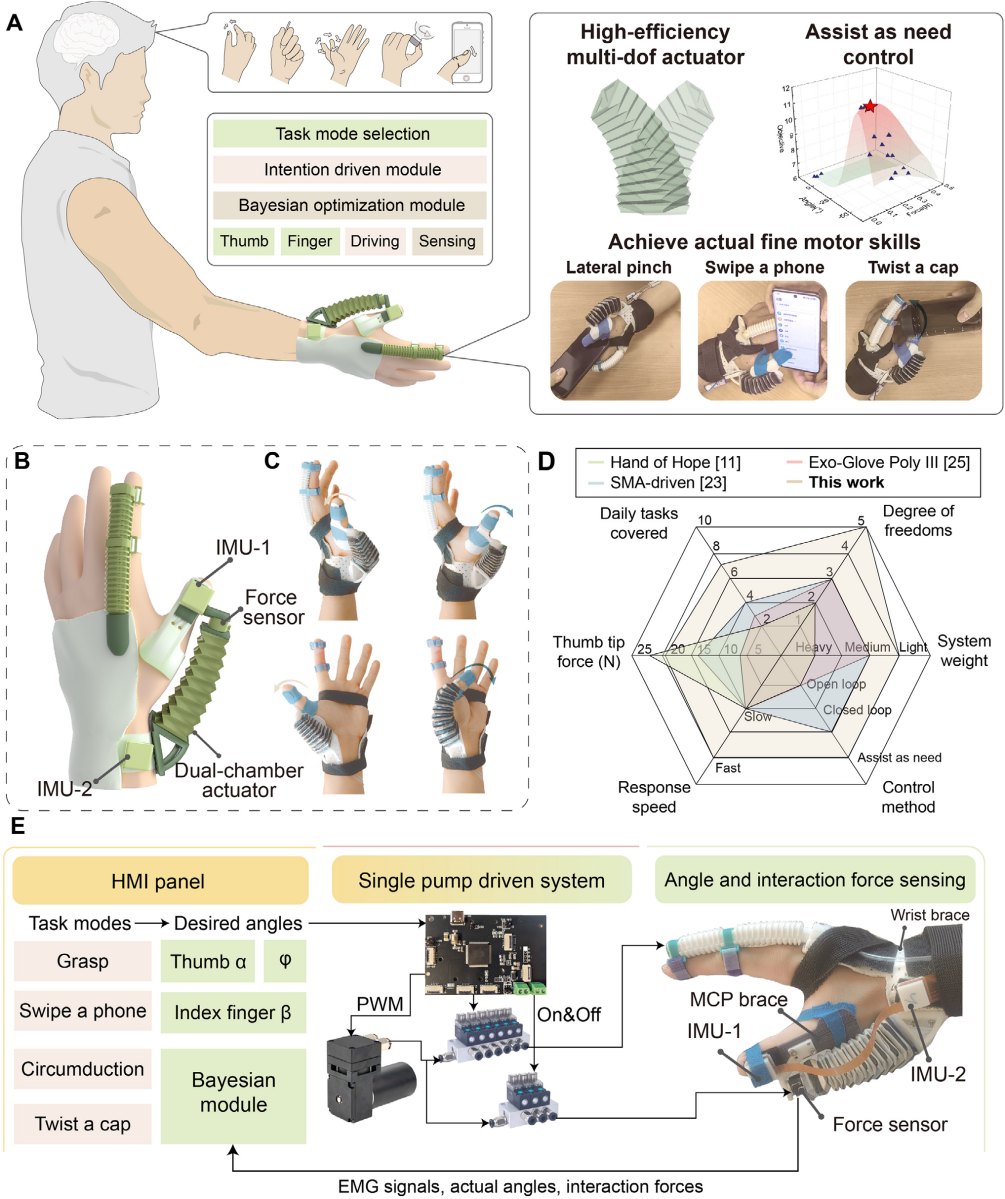
System of the portable soft robotic glove with fully functional thumb assistance. (a) An overview showing how the soft glove assists stroke survivors in performing various daily activities, such as lateral pinching, swiping a phone, and twisting a cap. The core innovation is the multi‐degree‐of‐freedom single actuator and the assist‐as‐needed control method deployed on it. The user first selects the desired task mode via a smartphone app, after which the system detects the user's intention to perform the task. The optimization module then assists the movements of the thumb at the lowest level of assistance, maximizing user involvement to achieve the tasks using the actuation and sensing components. (b) Schematic illustration of the soft robotic glove with an enhanced thumb component utilizing the proposed dual‐chamber origami actuator. This design also incorporates a force sensor and inertial measurement units (IMUs) mounted on top and bottom end of the actuator to detect thumb motion and the interaction force between the thumb and actuator. The origami actuator is segmented by planes that intersect the parallel edges on both sides. (c) Photos of the soft robotic glove demonstrating thumb adduction, abduction, extension, and flexion. (d) Comparison of daily tasks covered, degrees of freedom, thumb tip force, response speed, control method, and system weight among typical robotic devices that assist hand movement, the Hand of Hope [14], SMA‐driven glove [23], Exo‐Glove Poly III [25], and this work. Our soft glove demonstrates superior performance compared to these methods. (e) The working principle of the soft glove, in which commands for the desired task are sent to the human‐machine interface (HMI), processed by the control circuit, and executed by opening and closing valves to deform the target thumb and fingers. The actual bending angle of the thumb is measured by the relative postures of two IMUs–one mounted on the fingertip and the other on the wrist. The assistive force, measured by the force sensor, is fed to the optimization module for closed‐loop control.

In summary, at the mechanical level, there are currently few gloves that adequately address the tasks required for daily life as shown in Note S1 and Table S1. Furthermore, as the DOFs increase, the design of the control box becomes increasingly sophisticated. Previously reported high‐DOF hand gloves have seldom reported portable driving methods, let alone demonstrated compatibility with advanced control techniques as shown in Table S1, limiting their integration into daily life. These limitations render soft robotic gloves ineffective for assisting with complex, dexterous fine motor skills in everyday situations, classifying them as devices for controlled environments. This paper proposes an improved soft robotic glove featuring a dexterous thumb assistance, as illustrated in Figure 1a. It incorporates a soft continuous‐segmented dual‐chamber origami actuator that actively assists with bidirectional motion for the CMC joint and bending of the metacarpophalangeal (MCP) joint, along with an extension plate for the interphalangeal (IP) joint. This design allows users to perform various daily tasks, such as pinching, swiping a phone, and twisting a cap. The glove integrates an adaptive, “assist as needed” control method and a portable driving system, making it usable in various scenarios. The thumb actuator, shown in Figure 1b, is a dual‐chamber origami actuator made from soft, non‐stretchable material. By using a continuous segmentation method, the actuator is divided into two continuous‐segmented chambers without compromising its deformation. This design maximizes assistive force, range of motion (ROM), and efficiency ever reported, enabling bidirectional bending when differential pressures are applied to the two chambers, as illustrated in Figure 1c. This establishes the mechanical foundation of the glove, making it the first to support most daily activities for stroke survivors during task‐oriented training. The system allows for user‐friendly interaction, and its single‐pump driven addresses the compactness issue associated with increased DOFs. Furthermore, it is compatible with assist‐as‐needed control techniques based on Bayesian optimization, achieving tasks with lowest assistive level to encourage the maximized involvement of users. Overall, the system achieves superior performance compared to other solutions, as shown in Figure 1d,e and in Note S1. The proposed system represents and pushes a significant advancement in hand assistive and rehabilitation technology, offering a practical solution for restoring functional hand movements in both task‐oriented training and daily life scenarios.

## 2 Results

### 2.1 Continuous‐Segmented Dual‐Chamber Origami Actuator

**FIGURE 2:**
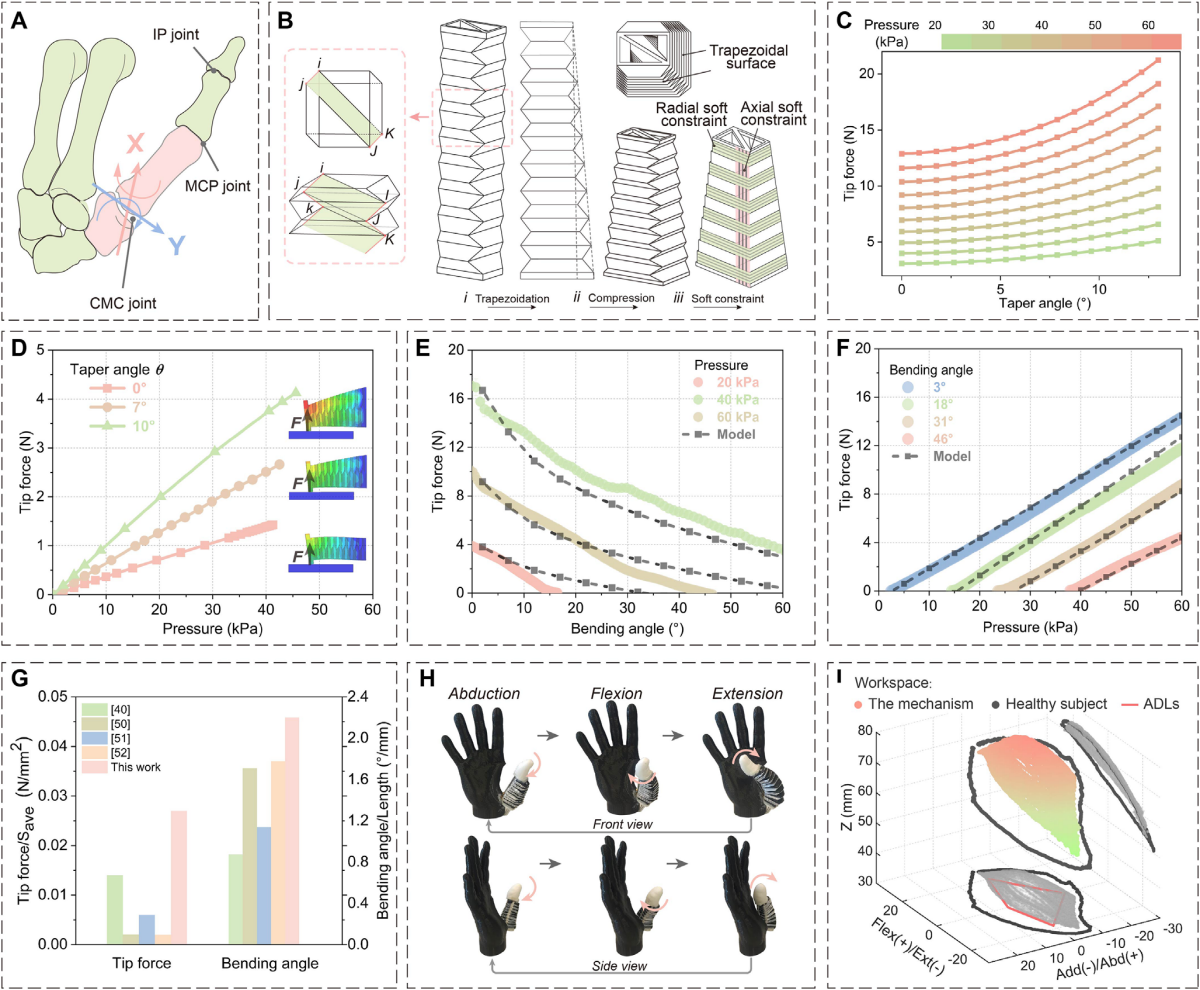
Design concepts of the continuous‐segmented dual‐chamber origami actuator (a) A biomechanical schematic illustrating the carpometacarpal (CMC), metacarpophalangeal (MCP), and interphalangeal (IP) joints. (b) The continuous segmentation method for a single origami actuator and the trapezoidal design process. (c) The modeled relationship among tip force, taper angle, and bending angle. (d) Finite element method simulations of the relationship among taper angle, tip force, and pressure. (e) Modeled and experimental relationships between tip force and bending angle. (f) Modeled and experimental relationships between tip force and pressure. (g) Comparison of the dual‐chamber actuator with other methods. (h) Model tests demonstrate that the dual‐chamber actuator enables the model thumb to achieve circumduction motion. (i) The actuator's range of motion encompasses the typical range of thumb movement for daily activities.

The dexterous core of the hand originates from the thumb, with the major joint being the CMC joint. This joint is illustrated by two vertical axes representing abduction/adduction and flexion/extension movements [32], as depicted in Figure 2a. The MCP joint is a single DOF joint. The two joints can be strategically assisted by a single two‐DOF bending actuator to improve compactness.

The ROM for the CMC joint is as follows: flexion (21.7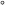), extension (19.5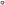), and abduction/adduction (0‐51.1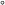) [33, 34, 35]. However, a range of ‐8 to 16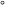 for abduction/adduction has been identified as sufficient to cover 100% of ADLs [26]. The force exerted by the thumb is also a crucial factor in facilitating movements related to ADLs. It is found that the torque required for thumb flexion, extension, abduction, and adduction ranges from 0.06 to 1.4 Nm [36]. During typical ADLs, the force at the thumb tip ranges from 3 to 9 N [37]. Hence the thumb part of the glove must fulfill these requirements.

Recent advancements in soft actuators enable multi‐directional bending and extension/contraction through parallel arrays. For instance, three 120
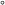
‐symmetric unstretchable material bending‐based bellows achieve two‐DOF motion [38], while four origami actuators enable three‐DOF (axial/bidirectional) movement [39]. However, integrating multiple actuators requires a large volume, whereas to achieve compactness, the cross‐sectional area of each chamber may be compromised, resulting in insufficient assistive force. Another approach enhances compactness by adding internal partitions to a single large stretch‐based actuator, resulting in dual‐chamber [40], three‐chamber [41], and four‐chamber soft actuators [42]. Unstretchable material bending‐based soft actuator, such as bellow or origami actuator has its advantages over the large‐stretch based actuator such as the high‐upper bound pressure bearing ability [43] to ensure enough assistive force. However, its low stretching nature hinders the segmentation of the actuator, i.e., the wrong segmentation hinders the movement of the original motion and causes the ROM to be less and even the failure of the structure. Hence there requires a continuous segmentation method to divide the origami actuator into a multi‐chamber one not to affect the deformation of the actuator. To achieve 2‐DOF bending at the CMC joint and bending motion at the MCP joint, a segmented three‐chamber design, not two‐chamber is the most intuitive choice. However, increasing the number of chambers reduces the cross‐sectional area of each chamber, resulting in decreased output force for the same pressure or volume [44]. Consequently, the generated force may be inadequate to overcome the inherent stiffness of the CMC joint, restricting its application in practical wearable scenarios. Hence, a segmented dual‐chamber origami actuator with constraint is chosen for the actuation.

To address the issue of internal partitions affecting the extension and contraction of the actuator and to ensure motion continuity between the internal partition and the external surface, this paper proposes a chamber segmentation method performed at the diagonal positions of the actuator, based on the folding theory of origami structure [45, 46], as shown in Figure 2b. A basic quadrilateral origami structure is used, ensuring that the angles of the mountain creases match those of the valley creases, resulting in parallel intersection lines of the staggered diagonal creases. Specifically, in Figure 2b, edges ij and IJ are parallel, as are edges jk and JK. Using edges ij and IJ as references, a sketch plane is established, connecting points i and I, and points j and J to generate the spatial rectangular plane ijJI. The same method is applied to create another spatial rectangular plane jkKJ using edges jk and JK. Finally, a continuous internal partition is generated through an array operation, resulting in the dual‐chamber structure. More detailed continuation proofs are provided in the Note S2, where the comparison with other segmentation method is also compared. It is recognized that a higher length‐to‐side length ratio leads to increased instability, significantly reducing the tip force of soft actuators [47], whereas a larger taper angle improves structural stability, resulting in greater tip force [48, 49]. Therefore, the proposed actuator is designed in a trapezoidal origami configuration, as shown in Figure 2b. A mathematical model is proposed in Note S3 to describe the relationships among taper angle, bending angle, and tip force. To enhance the pressure‐bearing capacity and enable a higher output sufficient for assisting thumb movement, a soft constraint is applied to the actuator, as shown in Figure 2b. The tested constrained dual‐chamber actuator can withstand pressures of up to 150 kPa and support a weight of 60 N at its end. As shown in Figure 2c, the tip force exhibits a significant increase when the taper angle is enlarged from 0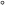 to 15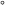. This increase is attributed to the improved structural stability under larger taper angles, thereby optimizing the energy conversion efficiency from pneumatic input to tip force output. The model also shows that the tip force reduces with the increasing of initial height as shown in Figure S1, emphasizing that the parameters require customization for certain user. Finite element method (FEM) model also shows the same trend that increasing the taper angle, the tip force increases a lot at the same pressure, as shown in Figure 2d. The parameters used for FEM are shown in Table S2.

To measure the bending angle under various pressures, the inertial measurement unit (IMU) is installed on the top of the actuator. As shown in Figures S2 and S3, only inflating one chamber to 60 kPa, the bending angle can reach 102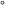, while a dual‐chamber pressure coordination strategy (simultaneous inflation of one chamber and vacuum of the other) significantly increased the bending angle to 143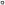. This enables the actuator to exceed the natural range of human thumb motion under no‐load conditions, thereby establishing a foundation for multi‐DOF assistive applications.

The mechanical characteristics under different bending angles and pressures are analyzed. The experimental results are shown in the Figure 2e,f conforming to the mathematical model results. The results show that even under the maximum angle of the CMC joint, the actuator can still maintain a tip force output of about 4 N at only 60 kPa input. This performance meets the requirements of joint assistance.

Figure 2g is a comparison of the bending angle and tip force with other types of dual‐chamber actuators [40, 50, 51, 52]. More detailed comparisons are shown in Table S3. Due to the varying dimensions of different actuators, as well as the tip force and bending angle are proportional to the cross‐sectional area ($S_{ave}$) and length of the actuator, respectively, the two parameters are normalized by area or length for fair comparison [53]. The results demonstrate that the improved origami has the highest normalized tip force and normalized bending angle compared with other large‐stretch based and unstretchable material bending‐based methods. After a stroke, thumb stiffness from spasticity makes the output force of actuator crucial: the actuator must overcome this stiffness and still have enough force to assist tasks. Yet the actuator cannot be large due to the thumb's limited surface area. Simply increasing volume or cross‐sectional area would compromise the glove design and hinder natural movement. This is another reason why we compare existing works using normalized tip force.

Hysteresis is a universal phenomenon in practical use of the soft actuator [54]. The pressure‐bending angle and pressure‐tip force hysteresis loop are tested, as shown in Figure S4. It can be concluded that the hysteresis of the pressure‐tip force and that of the pressure‐bending angle are about 13% and 18%, respectively. The lifetime of the proposed actuator is shown in Figure S5. After 5850 cycles, the breakage appears and the bending angle reduces by 4.2%. The dual‐chamber actuator can be made using the manufacturing method shown in Experimental Section.

To demonstrate the potential range of motion of the dual‐chamber actuator, a model hand is constructed with a universal joint at the CMC joint position to represent this joint. The dual‐chamber actuator is applied at the same position when wearing, as shown in Figure 2h. Two tests are conducted: first is the circumferential rotation test as illustrated in Figure 2i. To quantitatively evaluate the workspace of the proposed mechanism, 3D spatial coordinates of the mechanism's end effector are captured in real time using the NOKOV motion capture system. A healthy male subject with 180 cm height is recruited, conducting the circumferential movement to compare the range data with that of the model hand directly. The range shown in Figure 2i is similar to that of a database from 28 subjects [55]. Analysis reveals that the mechanism achieves 86.8% coverage of the natural thumb motion range, as shown in Figure 2i covering the requirements of ADL, indicating its feasibility in terms of motion range.

The second test is the phone swiping test, which represents one of the most important activities in modern life. By fine‐tuning the pressure in the two chambers, the mechanism replicated thumb movement while swiping on phones, as shown in Figure S6 and Movie S1. Experimental results indicate a range of 20 mm for each swiping up and 18 mm for each swiping down, demonstrating the mechanism's efficacy.

### 2.2 Ergonomic Design of the Thumb and Finger Part

To maximize the assistive effect of the dual‐chamber actuator, the design of the anchor must be carefully considered, as previous research on the human‐exosuit interface suggests [56]. Specifically, lower stiffness at the interface or anchor causes more energy absorbed by the interface. However, while high‐stiffness materials can be employed to increase stiffness, the DOFs of CMC joint should not be restricted by inhibiting the deformation of the thenar eminence [57]. Additionally, the thumb tip must remain unobstructed to ensure proper task performance. Therefore, as shown in Figure 1b,e, top end of the actuator is fixed to the proximal phalanx between the IP and MCP joints of thumb through a MCP brace whereas the bottom end is secured to a wrist brace as shown in Figure 1b and Figure S7a,b. The MCP brace is a flexible plate that covers the IP joint and MCP joint, secured to the thumb with a muscle patch to provide a firm connection between the actuator and the thumb. Notably, the IP joint is fixed at an extended angle using the muscle patch by the MCP brace to overcome the increased muscle tone in IP joint. The active assistance of the IP joint was considered but not implemented, as to finish the tasks specified in the paper does not require the assisting of IP joint actively. In addition, elongating the actuator would reduce the tip force due to the increased instability. Maintaining the same tip force requires a lar ger bottom area, increasing the bulkiness of the system. Some stroke patients, while exhibiting spasticity, retain residual volitional control. This flexible MCP brace accommodates active bending motion through intentional subject engagement, following the same design concept as the previous successful practice [58]. The MCP joint remains movable to let the dual‐chamber actuator drives flexion/extension around the MCP brace and prevent hyperextension when the thumb is pushed by the actuator, as shown in Figure S7a,b.

During thumb adduction assisted by the actuator, the tip force is transmitted from the thumb to the wrist, as shown in Figure S7c. At certain postures, the stiffness of the CMC joint can exceed that of the wrist joint, leading to wrist extension instead of thumb adduction, as shown in Figure S7c. To address this, a wrist brace covering the back of the hand and wrist brace is designed to constrain wrist movement, as shown in Figure S7d. Research indicates that optimal grip strength is achieved when the wrist is positioned at 30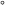 [59]. Therefore, the wrist brace maintains the wrist in this extended position for optimal assistive effect.

Thermoplastic plates are selected as the material for the brace structure. The plates are heated in hot water until they become flexible and are then carefully molded onto the subject's hand after partial cooling to ensure complete anatomical conformity and comfort. Once fully cooled and solidified, the structure is trimmed with fully exposed CMC joint and assembled with the dual‐chamber actuator, secured using Velcro straps for a comfortable, wearable fit, as shown in Figure 1b,e. One elastic Velcro strap secures the wrist joint, while the other crosses the palm to stabilize the hand. This method allows for customized adjustments based on individual anatomical variations, and the individualized design of the dual‐chamber actuator is detailed in Note S4 to tailor the actuator parameters according to user requirements.

The finger actuator is adapted from previous work, utilizing a single‐chamber actuator with a steel plate in the chamber [58, 60], as shown in Figure 1b. This design passively extends the spastic finger when deflated and helps flex the finger when the chamber is inflated, making it suitable for assisting stroke patients with a Modified Ashworth Scale (MAS) score [61] of 3 or lower. The design around thumb IP joint is also inspired by the success of this practice.

### 2.3 Control System, Deployed Air Circuit, and Low Level Control Method

**FIGURE 3:**
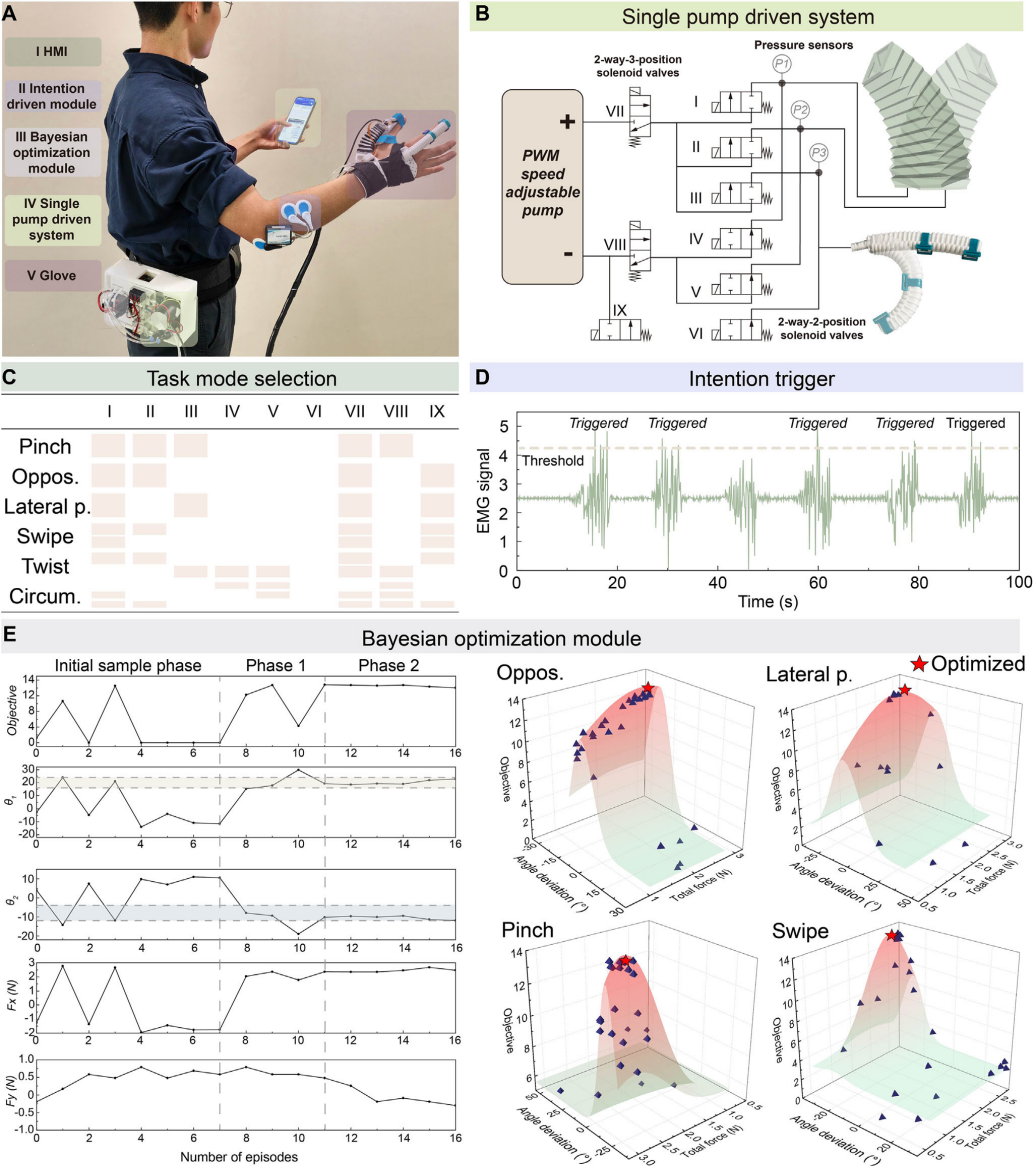
Schematic of the overall control system. (a) The control system consists of a human‐machine interface app installed on a smartphone. The user chooses the task they wish to perform in the app, and the intention‐driven module detects their intent to execute the Bayesian optimization algorithm on the single pump‐driven system, utilizing the glove to assist with the task. (b) Components of the single pump‐driven system. (c) The on/off logic of the valves in the single pump‐driven system for conducting different tasks, with pink blocks indicating open valves and other conditions indicating closed valves. (d) The intention trigger logic based on EMG signals. (e) Controlled parameter changes during subject validation. The optimization comprises three phases: the initial sample phase, where the system samples the preset motion to obtain the assistive force at different angles; phase 1, where only the bending angle of the thumb is optimized to allow the user to quickly achieve the task without losing confidence, thereby increasing the speed of convergence; and phase 2, where the system not only ensures the task is accomplished but also minimizes the assistive force. The right side illustrates the landscape of the optimization objectives during task execution in phase 2, showing that all objective functions reach a peak, indicating the achievement of the desired task while maintaining the lowest level of assistive force, in accordance with the “assist‐as‐needed” control concept.

The overall control system is shown in Figure 3a. The user wears the glove on the impaired side while holding a smartphone with the healthy side. An “I. Human‐Machine Interface (HMI)” application is installed on the smartphone. The user selects the task they wish to perform within the application, and then the “II. Intention‐Driven Module” polls the user's intent to execute the “III. Bayesian Optimization Algorithm” on the “IV. Single Pump‐Driven System” mounted on their back, assisting the users' movements with the glove.

The core control hardware, the IV. single pump‐driven system shown in Figure 3b, consists of one pulse width modulation (PWM) speed‐adjustable pump and nine solenoid valves. Among these, seven are 2‐way, 2‐position solenoid valves, while the remaining two are 2‐way, 3‐position valves, labeled I‐IX in Figure 3b. Valves I and IV control the pressurization and vacuum for chamber 1 of the dual‐chamber actuator, while valves II and V manage the same for chamber 2. Similarly, valves III and VI regulate the inflation and deflation of the finger actuator. Valves VII and VIII ensure that when the system is inactive, pressurized air in the air pipes can flow to the ambient atmosphere, preventing obstruction of the pump during the next activation. Valve IX is used because the pressure required for the finger actuator is greater than that for the dual‐chamber actuator; thus, the pressurization time for the finger actuator is longer and is delivered through valve IX. The logic for opening and closing the valves for different tasks is illustrated in Figure 3c.

It is important to note that pinch, opposition, and lateral pinch movements consist of single elemental movements; therefore, once these movements are completed, the system returns to its initial conditions. In contrast, swipe, twist, and circumduction movements involve multiple elemental movements. For example, the swipe task requires the thumb to first contact the screen and then swipe up or down. The twist task necessitates the thumb to flex while the finger extends, followed by the opposite action. Circumduction starts with thumb abduction, proceeds to flexion, and concludes with adduction. Consequently, the valve logic changes once for single elemental movements but multiple times for tasks involving several elemental movements.

The intention trigger module is illustrated in Figure 3a,d and features two surface electromyography (EMG) sensors mounted on the forearm to detect muscle signals for finger flexion and extension, thereby triggering the system. When the signal exceeds a specified threshold, the system activates a low‐level optimization algorithm on the single pump‐driven system.

The glove is initially used for rehabilitation training, hence the low‐level control method must align with the training protocol. As previously mentioned, single tasks can be divided into elemental movements, with each task requiring the user to perform these movements multiple times. Consequently, the system undergoes several cycles to adjust its parameters. Recent research indicates that assistive systems should provide the minimum level of assistance while maximizing user involvement in training for optimal rehabilitation effects, a concept known as the “assist as needed” (AAN) method [62, 63]. The control method must also accommodate individual variability. The human‐in‐the‐loop (HIL) approach, utilizing Bayesian optimization, addresses this variability and has gained attention, particularly for lower limb applications, where cyclic movements allow for optimization to minimize metabolic costs during walking [64]. However, reports on upper limb applications are limited.

During training, multiple repetitions of elemental movements create a cyclic behavior, making the HIL method applicable in this context with a training‐oriented cost function to achieve the AAN effect. As shown in Figure 1e, IMU and force sensors mounted on the thumb tip detect the bending angle and interaction force between the thumb and the actuator. According to the AAN method, the assistive force should be minimal while still enabling task completion, meaning the thumb must bend to a prescribed angle. Thus, the cost function is formulated as follows:

(1) $$J_{2}=\omega_{r}\cdot R_{angle}-\omega_{f}\cdot P_{force}$$

here, $\omega_{f}$ balances the minimization of force against angle maintenance, ensuring the system reduces assistance without compromising movement quality. $R_{angle}$ is the prescribed and preprocessed angle needed to complete one elemental task, measured by the IMU, while $P_{force}$ refers to the preprocessed assistive force measured by the force sensor. Detailed control methods are presented in Note S5.

Figure 3e illustrates the optimization process for one elemental movement. The first seven cycles are initial sample stage to collect the data to construct the probabilistic mapping. In the following five cycles of stage 1, the system conducts single‐objective optimization to quickly reach the required angle for task completion, building the user's confidence. After two cycles, dual objectives are optimized to minimize assistive force while maintaining the prescribed angle. The right part of Figure 3e shows the changes in the objective function value with thumb angle and assistive force. Each task displays a peak, indicating the lowest assistive force while successfully achieving the task, conforming to the concept of AAN.

### 2.4 Subject Validation

The following inclusion‐exclusion criteria are applied for recruiting subjects to participate in the on‐body tests: (a) patients with chronic stroke (at least six months post‐onset) presenting with motor paralysis due to unilateral brain injury; (b) adequate cognitive capacity to follow simple instructions and understand the training content and purposes; (c) ability to sit up for at least 45 min; and (d) a MAS score for finger extension of ≤3. The exclusion criteria include the presence of significant language barriers that hinder effective communication or comprehension of research instructions. The study is approved by the Joint Chinese University of Hong Kong–New Territories East Cluster Clinical Research Ethics Committee (The Joint CUHK NTEC CREC). Ref No. CREC 2020.698 and complies with the Declaration of Helsinki. All subjects provided written informed consent before the experiments. According to the stratification method using FMAue [65], where scores of 0–28 indicate severe impairment, 29–42 indicate moderate impairment, and 43–66 indicate mild impairment, three chronic stroke subjects with varying impairment levels from severe to mild are recruited, and their demographic information is presented in Table S4.

**FIGURE 4:**
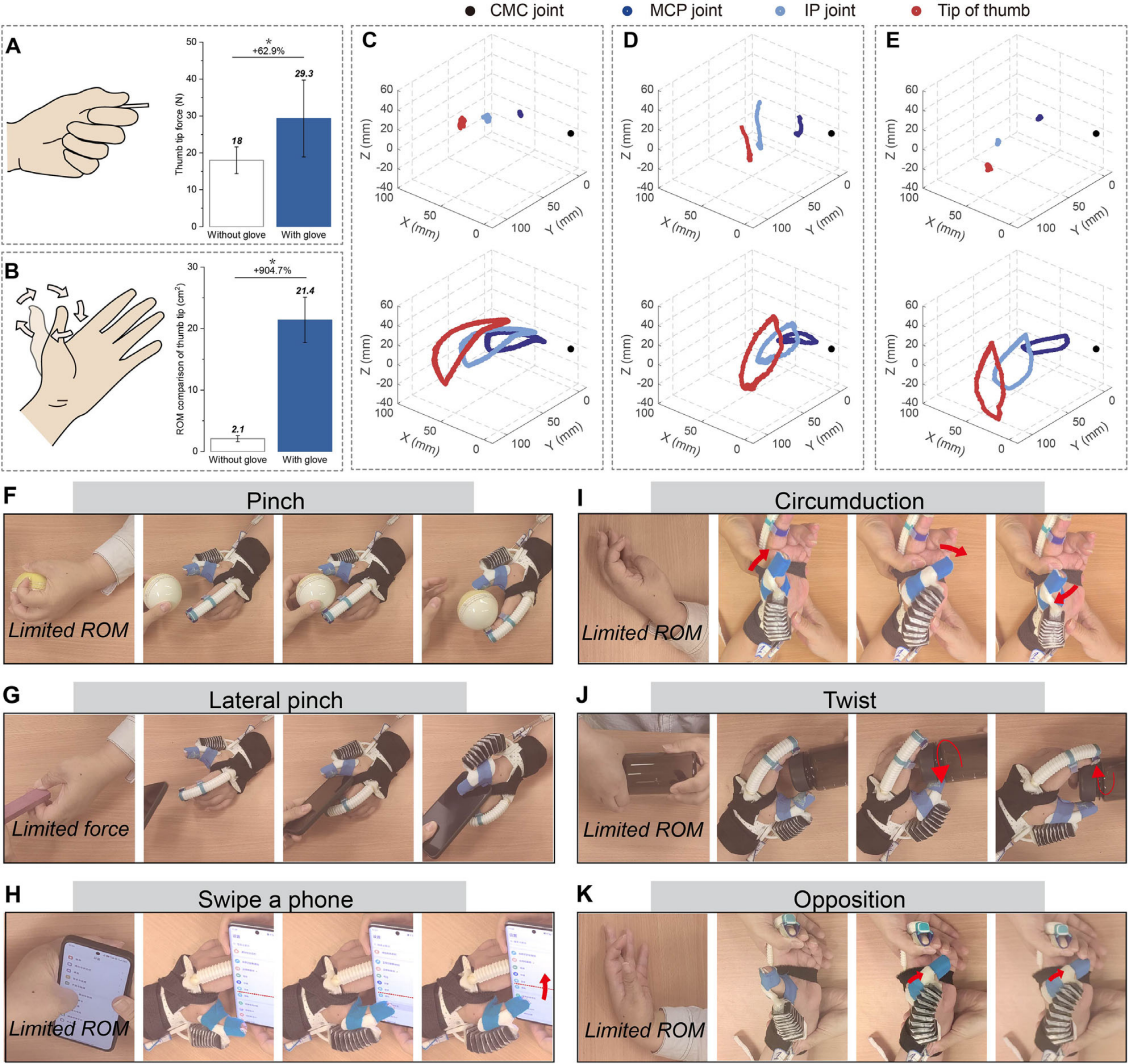
Subject test results. (a) Wearing the glove increases lateral pinch force by 62.9% compared to not wearing the glove. (b) Wearing the glove increases the range of motion for thumb circumduction by 904.7%. Range of motion comparisons for subjects S1 (c), S2 (d), and S3 (e) between wearing the glove and not wearing it. (f) Photos of test activities: pinch (f), lateral pinch (g), swiping a phone (h), circumduction (i), twisting a cap (j), and opposition (k) on subjects.

Similar to the lateral pinch task, subjects are instructed to press against a high‐precision force transducer (Baseline Muscle Test Pinch Dynamometer) as shown in Figure 4a with their thumb under two conditions: glove‐assisted and without the glove. The peak force values are recorded three times and averaged. As shown in Figure 4a, the soft robotic glove demonstrates an average enhancement of 62.9% in thumb tip force compared to the condition without the glove, exceeding the requirements for daily life use.

The ROM test is conducted using four markers placed at key anatomical landmarks of the subjects' hands: the thumb tip, IP joint, MCP joint, and CMC joint. As shown in Figure 4b, the experimental results revealed a significant increase in the thumb's ROM with the soft robotic glove compared to the without wearing condition. As shown in Figure 4b, the circumferential motion area exhibited a substantial enhancement–over nine times–when assisted by the soft robotic glove, compared to the minimal range observed without it. Figure 4c–e show the range of motion changes of the subject S1, S2, and S3. It is worth noting that the subject S3, although with the highest MAS scores, her ROM of thumb also increases about nine times, showing the sufficient driving force of the thumb part for rehabilitation training.

To validate the efficiency of the proposed soft robotic glove as a rehabilitation/assistive device, subjects are required to perform the following tasks under two conditions: with (assisted condition) and without (without wearing condition) the soft robotic glove:

(a) Pinch: pinch differently sized wooden blocks (25–70 mm), a 70 mm‐diameter sphere; The difficulty is gradually increased until they cannot pinch a larger size.

(b) Lateral pinch: holding a wooden strip (10 mm thick) or a smartphone;

(c) Opposition test: thumb‐to‐little‐finger opposition;

(d) Screen operation: swiping up or down the screen of smartphone;

(e) Thumb circumduction: full‐range circular movement of the thumb;

(f) Rotary manipulation: twisting bottle cap clockwise and counterclockwise (55 mm diameter) using the coordination of thumb and index finger, rather than relying on wrist pronation and supination.

As shown in Figure 4f–k and Movie S1, all tasks are performed under two conditions. The results demonstrate that the proposed soft robotic glove significantly improves motor function in stroke patients, validating its efficiency as a rehabilitation/assistive device.

## 3 Conclusion

This paper proposes a portable, dexterous, thumb‐enhanced soft robotic glove that utilizes a dual‐chamber origami actuator to drive the full degrees of freedom around the thumb CMC and MCP joint, while passively keeping the IP joint extended, thereby facilitating various daily tasks for stroke rehabilitation training. This design effectively addresses the long‐standing challenge of providing an effective thumb‐driven method. The dual‐chamber origami actuator can achieve up to 18 N of tip force, nearly double the force required for daily activities. A mathematical model based on the pseudo‐rigid body method is developed to explain the effects of taper angle, original length, and pressure on the tip force, guiding the actuator's design. Using a model hand to test the thumb part, results show that the proposed design covers approximately 86.8% of the CMC joint range of motion of a healthy subject, covering the daily activities requirements. A control system composed of an HMI, intention‐driven module, and Bayesian optimization module is deployed on a portable single pump‐driven system to provide rehabilitation and assistance in non‐structured scenarios, achieving assistance only when the user intends and implementing an “assist as needed” control algorithm. By recruiting three stroke subjects with varying levels of impairment, the most common daily tasks are successfully performed by all participants. These tasks included pinch, lateral pinch, thumb circumduction, phone swiping, cap twisting, and opposition. The pinch force of the thumb increased by 62.9% compared to the unassisted condition over the requirements of daily use, and the range of motion of the thumb improved nine times, demonstrating the efficacy of the system. The proposed system represents a significant advancement in hand rehabilitation medicine, offering a practical solution for dexterous, task‐oriented training aimed at restoring functional hand movements and assisting in daily life scenarios. In future studies, a two‐arm clinical trial will be conducted to compare thumb‐specific dexterous training combined with hand opening and closing training versus hand opening and closing training alone, and to assess whether thumb training can effectively improve hand function.

## 4 Experimental Section

### 4.1 Manufacturing Method of the Actuator

Figure S8 illustrates the actuator's fabrication process, primarily using 3D printing and injection molding. First, a solid prototype is produced via 3D printing. Next, silicone molding created a mold for the actuator, with liquid silicone poured in and allowed to cool and solidify. After removing the solid prototype, the mold was resealed, and a pouring port was added based on material fluidity and workpiece dimensions. Three rubber components (DPI 8400, YinKe Tech. Ltd., China) were mixed to achieve the desired hardness, and a vacuum infusion machine evacuated air from the mold before injecting the mixed material. Once filled, the mold was placed in an oven to accelerate curing before demolding. Finally, a compression device compresses the actuator, which was then tempered in a constant temperature chamber at 220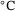 for half an hour, yielding the final actuator. It was worth noting that the actuator is first made in an extended state and then compressed into a contracted state before solidifying. This approach was necessary because the angle between each plate in the contracted state was too small (20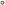 shown in Table S5), making the injection molding process difficult.

### 4.1 Characterization Setup

The bending angle–pressure, force‐pressure relations through a series of quasi‐static experiments were characterized. Pressure data was recorded by pressure sensor (XGZP6857A, ‐100‐300 kPa, CFSensor Inc), while bending angle was recorded through an IMU module (JYP61, WitMotion). And a microcontroller (Arduino Mega 2560) was responsible for real‐time signal acquisition. Tip force was measured using a force sensor (DYMH‐103, DAYSENSOR).

## Author Contributions

Conceptualization: D.X., R.K.T. Methodology: D.X., Z.W. Validation: D.X., Z.W. Formal analysis: D.X., Z.W. Investigation: D.X., Z.W. Resources: D.X., Z.W., Z.Y., X.S., Y.S., H.W., J.L., H.L., and T.L. Data curation: D.X., Z.W. Writing – original draft: D.X. and Z.W. Writing – review and editing: D.X., Z.W., and R.K.T. Visualization: D.X. and M.L. Supervision: R.K.T. Project administration: R.K.T. Funding acquisition: R.K.T.

## Funding

This work was supported by HKSAR Research Grants Council General Research Fund, Grant No: 14204925. C.M. would like to thank the Hong Kong Jockey Club Charities Trust for supporting the JC STEM Lab of Nature‐Inspired Precision Medical Engineering.

## Conflicts of Interest

D.X., Z.W., and R.K.T. are the inventors on a provisional patent application filed relating to this work by The Chinese University of Hong Kong. The other authors declare that they have no conflicts of interests.

## Supporting information


**Supporting File 1**: advs76275‐sup‐0001‐SuppMat.pdf.

**Supporting File 2**: advs76275‐sup‐0002‐MovieS1.mp4.

## Data Availability

All data are available in the main text or the supplementary materials.
