# Supplementary material for: A Portable Soft Robotic Glove with Fully Functional Thumb Assistance for Complex Dexterous Fine Motor Skills
Source: Adv Sci (Weinh). 2026 Jun 26:e76275. Online ahead of print. doi: 10.1002/advs.76275 (PMC13336747; doi:10.1002/advs.76275)
Supplement: Supplementary file 1 — Supporting File 1: advs76275‐sup‐0001‐SuppMat.pdf. [file ADVS-9999-e76275-s002.pdf]

Supplementary Information for: A Portable Soft Robotic Glove with Fully Functional Thumb Assistance for Complex Dexterous Fine Motor Skills

*Disheng Xie† Zhen Wang† Zhongping Ye Minghao Liu Xiangqian Shi Shuk Fan Tong Jianbin Liu Chuanbin Mao Thomas Leung Haitao Liu Raymond Kai-yu Tong**

Disheng Xie and Zhen Wang share the same contributions to this paper.
Corresponding author: Raymond Kai-yu Tong.

Disheng Xie, Zhen Wang, Zhongping Ye, Minghao Liu, Xiangqian Shi, Shuk Fan Tong, Chuanbin Mao, and Raymond Kai-yu Tong
Address: Department of Biomedical Engineering, the Chinese University of Hong Kong, Hong Kong SAR, China
Email Address: xiedisheng@link.cuhk.edu.hk,zhenwang@link.cuhk.edu.hk, zpye@link.cuhk.edu.hk, xqshi@link.cuhk.edu.hk, fannytong@cuhk.edu.hk, cmao@cuhk.edu.hk, kytong@cuhk.edu.hk
Jianbin Liu and Haitao Liu
Address: Department of Mechanical Engineering, Tianjin University, China
Email Address: jianbin_liu@tju.edu.cn, liuht@tju.edu.cn
Thomas Leung
Address: Faculty of Medicine, the Chinese University of Hong Kong, Hong Kong SAR, China
Email Address: drtleung@cuhk.edu.hk

Note S1. Systematic comparison of existing robotic glove systems

Robotic devices that assist hand movement can be categorized into the following groups based on their drive and transmission methods: exoskeletons using motor drives with rigid links to transmit force, and soft robotic gloves, which can be further divided into motor-cable-driven types, artificial muscle-driven types, and soft bending actuator-driven types. Their key characteristics are systematically summarized in Table S1.

Some hand exoskeletons are designed without a thumb, focusing exclusively on the other four fingers [1–3]. Other systems, such as the Hand of Hope [4] and RobHand [5,6], provide assistance with the flexion and extension of both fingers and the thumb, facilitating basic hand opening, closing, and grasping functions [7–10]. Improved thumb exoskeletons with multiple degrees of freedom (DOF) have been developed [11–15], enabling abduction and adduction movements. However, their large volume and high weight impede their use in task-oriented training and daily activities, while poor compliance restricts natural finger movement, affecting voluntary actions.

In contrast, soft robotic gloves are increasingly favored due to their lightweight design, natural joint movement, and comfortable wearing experience. Motor-cable-driven types, such as the Exo-Glove Shell [16] and FLEXotendon II [17], assist with finger extension and flexion. Some designs [18–20] also support thumb abduction, adduction, extension, and flexion. As shown in Table S1, most of these systems rely on open-loop control and lack force feedback. Due to the high DOFs of such gloves and the challenges of routing the pulling cables between the palm and thenar eminence to effectively achieve thumb opposition (the motion assisting the thumb toward the little finger), the tasks they can perform are limited. Furthermore, increasing the degrees of freedom inevitably raises the number of cables and motors, leading to a higher system weight.

Artificial muscle-driven systems have been explored as alternatives to motors. For instance, soft robotic gloves driven by shape memory alloys (SMA) [21, 22] and those using filament artificial muscles have been developed [23–25]. These devices can assist with thumb and finger flexion, extension, or opposition movements. However, they face the significant drawback of relatively slow response speeds, which fail to meet the demands of daily use.

Both cable-driven and artificial muscle-driven systems share a common challenge: their actuation components, such as cables, must be mounted on the palm's surface. This configuration often interferes with object manipulation during functional daily tasks.

Soft bending actuator-driven types [26–30] have emerged with lightweight designs, inherent compliance for natural movement, and self-alignment properties. Implementations like the Harvard glove [31] and the HandTasker [32] can assist with thumb and finger flexion and extension, demonstrating superior rehabilitative efficacy compared to rigid exoskeletons [33]. Despite these advantages, they still have limitations. Most cannot support multi-DOF thumb movements. Although some designs [34–36] can achieve thumb abduction, they cannot assist with both abduction and adduction, restricting complex daily tasks like twisting caps or swiping phones. Additionally, most of these devices rely on open-loop control and lack force feedback [37,38], compromising grasping precision, operational safety, and adaptive force modulation during interactions with the environment.

From Table S1, it can be concluded that the performance of the soft robotic glove proposed in this paper leads in most aspects, exhibiting the lowest weight, the highest number of thumb degrees of freedom, and significant thumb tip force. This enables it to accomplish a wide range of tasks using closed-loop and as-needed control methods.

Note S2. Kinematic compatibility demonstration

The main advantage of the proposed continuous segmentation method lies in its 'edge-edge-coupled' design. To validate this method ensuring continuity, a mathematical deduction is performed to demonstrate that the shape of the internal partition remains stable despite deformation of the external surface.

As shown in Fig. S9(a), a spatial coordinate system is established with the point E of the midplane as the origin, where the central axis of the actuator is parallel with $z - axis$. The upper surface of the actuator lies on the plane $z = h$, while the lower surface is positioned at $z = -h$. In the initial state, points D, E, L, K are coplanar. Assume the contraction or extension length of the actuator is $2s$, where $s > 0$ indicates extension and $s < 0$ indicates contraction. Due to the axial symmetry of the actuator, each points' coordinate of the partition are $D'(d_x + \Delta x, d_y + \Delta y, h + s)$, $L'(l_x - \Delta x, l_y - \Delta y, -h - s)$, $E(0, 0, 0)$, $K(k_x, k_y, 0)$ after deformation.

The reason of deformation obstruction is that when the outer surface deforms, the internal partition surface is stretched or bent. Hence to ensure compatibility with the original structure, coplanarity of points D, E, L and K must be preserved throughout actuation to ensure motion continuity.

The necessary and sufficient condition for four spatial points to be coplanar is that the scalar triple product of three vectors originating from one of these points equals 0. Taking the deformed point D' as the origin, three spatial vectors pointing to E, K, and L' (which directly characterize the relative positional relationships among the four vertices D, E, L and K) are constructed:

$$\overrightarrow{D'E} = (-d_x - \Delta x, -d_y - \Delta y, -h - s) = (-d'_x, -d'_y, -h - s) \tag{1}$$

$$\overrightarrow{D'K} = (k_x - d_x - \Delta x, k_y - d_y - \Delta y, -h - s) = (A - \Delta x, B - \Delta y, C) \tag{2}$$

$$\overrightarrow{D'L'} = (l_x - d_x - 2\Delta x, l_y - d_y - 2\Delta y, -2h - 2s) = (M - 2\Delta x, N - 2\Delta y, R) \tag{3}$$

where $A = k_x - d_x$, $B = k_y - d_y$, $M = l_x - d_x$, $N = l_y - d_y$, are fixed geometric parameters independent of the deformed displacement s and $d'_x = d_x + \Delta x$, $d'_y = d_y + \Delta y$,$C = -h - s$, $R = -2h - 2s$.

Then, the cross product of $\overrightarrow{D'K} \times \overrightarrow{D'L'}$ can be obtained as follows.

$$\overrightarrow{D'K} \times \overrightarrow{D'L'} = (B' \cdot R - C \cdot N', C \cdot M' - A' \cdot R, A' \cdot N' - B' \cdot M') \tag{4}$$

$$\overrightarrow{D'K} \times \overrightarrow{D'L'} = [-(2B' + N')(h + s), (-M' + 2A')(h + s), A'N' - B'M'] \tag{5}$$

where $A' = A - \Delta x$, $B' = B - \Delta y$, $M' = M - 2\Delta x$, $N' = N - 2\Delta y$.

Perform the dot product of $\overrightarrow{D'E}$ with the result, and extract the common factor $(h + s)$:

$$\overrightarrow{D'E}\cdot(\overrightarrow{D'K}\times\overrightarrow{D'L'})=(h+s)[2d'_xB'-d'_xN'+d'_yM'-2d'_yA'-A'N'+B'M']=(h+s)T \tag{6}$$

where $T=2d'_xB'-d'_xN'+d'_yM'-2d'_yA'-A'N'+B'M'$.

Each term in T can be calculated separately as follows:

$$2d'_xB'=2(d_x+\Delta x)(B-\Delta y)=2d_xB-2d_x\Delta y+2B\Delta X-2\Delta x\Delta y \tag{7}$$

$$-d'_xN'=-(d_x+\Delta x)(N-\Delta y)=-d_xN+2d_x\Delta y-N\Delta x+2\Delta x\Delta y \tag{8}$$

$$d'_yM'=(d_y+\Delta y)(M-2\Delta x)=d_yM-2d_y\Delta x+M\Delta y-2\Delta x\Delta y \tag{9}$$

$$-d'_yA'=-2(d_y+\Delta y)(A-\Delta x)=-2d_yA+2d_y\Delta x-2A\Delta y+2\Delta x\Delta y \tag{10}$$

$$-A'N'=-(A-\Delta x)(N-2\Delta y)=-AN+2A\Delta y+N\Delta x-2\Delta x\Delta y \tag{11}$$

$$B'M'=(B-\Delta y)(M-2\Delta x)=BM-2B\Delta x-M\Delta y+2\Delta x\Delta y \tag{12}$$

By summing up Eqs.(7) to (12) term by term, all terms containing Δx and Δy can be removed, thus deducing that $T=2d_xB-d_xN+d_yM-2d_yA-AN+BM$.

In the initial state, the contraction or extension length $s=0$, D, E, L and K has already achieved four-point coplanarity, hence the initial mixed product is 0.

$$\overrightarrow{DE}\cdot(\overrightarrow{DK}\times\overrightarrow{DL})=0 \tag{13}$$

$$h(2d_xB-d_xN+d_yM-2d_yA-AN+BM)=0 \tag{14}$$

Since $h\neq 0$, $T=2d_xB-d_xN+d_yM-2d_yA-AN+BM=0$.

Therefore, the mixed product $\overrightarrow{D'E}\cdot(\overrightarrow{D'K}\times\overrightarrow{D'L'})$ remains zero regardless of the value of the deformation s (whether extension or contraction). This proves that the four vertices of D, E, L and K are always coplanar during the structural deformation without spatial distortion, which satisfies the requirement of kinematic compatibility.

Comparison with other segmentation methods To further highlight the advantages of the diagonal segmentation using ‘edge-edge-coupled’ method, a comparison is made with ‘edge-surface-coupled’ method [39] where its chamber segmentation is realized through OO' // GJ // AD, $O'P'$ // GH, PP' // KH and edge-surface bonding (e.g., $O'P'$ is connected to external surface $DGHE$)).

For instance, when the actuator is extended, edges of the original structure (e.g., GH) move along an arc centered at edges of the middle plane (e.g., EH). However, due to the fixed-end constraints (O' is fixed on DG and P' is fixed on EH), parallel edges (e.g., $O'P'$) cannot fully follow this arc trajectory, leading to motion interference. In contrast, the diagonal segmentation method achieves 'edge-edge-coupled' without additional parallel constraints. Its motion trajectory can naturally adapt to the extension and contraction of the external structure, satisfying kinematic compatibility and achieves higher energy efficiency and deformation.

Note S3. Modeling and characterization of the dual-chamber actuator

To provide a theoretical basis for the design and performance analysis of the dual-chamber actuator, a mathematical model based on the pseudo-rigid body method (PRBM) is proposed [40, 41]. This model considers the continuum bending actuator as links that are interconnected, with the bending torque concentrated at the rotating joints between adjacent links.

First, the coordinate system is built as shown in Fig. S10, where the origin is set as O_O^0 at the vertex of the dual-chamber actuator, the Z-axis is the line vertical to the bottom surface and the X-axis is the line pointed from O_O^0 to the vertex on the opposite side of the bottom surface. Because the deformation of the dual-chamber actuator is limited by the soft constraint as shown in Fig. S10, the continuum dual-chamber actuator is considered as a series of interconnected links, where each link j is a part of the soft constraint between each pair of adjacent ridges. The torque M_j concentrated at the joint between Link-j and Link-(j-1) is from the volume change of the adjacent Unit-j divided from the dual-chamber actuator. Here, the unit is the volume enclosed by the Layer-(2j-2) and Layer-(2j-1) (e.g., Unit-2 is the volume enclosed by Layer-2 and Layer-3 as shown in Fig. S10). It is worth noting that the Unit-1 and Unit-N are the volume enclosed by Layer-1 and Layer-$2N$ respectively.

The geometric detailed parameters of the dual-chamber actuator are defined as follows:

- N: Total number of links, units, and the joint;
- a_1: Length of the top surface of the volume enclosed by Layer-1;
- b_1: Width of the top surface of the volume enclosed by Layer-1;
- h: Height of the single layer;
- θ: Taper angle of the trapezoidal dual-chamber actuator;
- d_1: Distance from the soft constraint to the centroid of the top surface of Layer-1;
- j: Index of links and units, $j = 1, 2, \ldots, N$;
- i: Index of layers, $i = 1, 2, \ldots, 2N$;
- q_j: Bending angle at joint between Link-j and Link-(j-1) (with q_N representing the angle between Link-N and the Z-axis);
- ΔV_j: Volume change of Unit-j.
- M_j: Torque at the joint corresponding to Unit-j, induced by the volume change ΔV_j of Unit-j.

Given the overall trapezoidal configuration, the top surface dimensions of the volume enclosed by each layer (length a_i, width b_i) and the distance d_i from the soft constraint to the centroid of the top surface of the volume enclosed by Layer-i are derived as follows:

$$\begin{aligned} a_i &= a_1 + (i-1)h\tan\theta \\ b_i &= b_1 + (i-1)h\tan\theta \\ d_i &= d_1 + (i-1)h\tan\theta \end{aligned} \tag{15}$$

Here, a_i and b_i are the length and width of the top surface of the volume enclosed by Layer-i($i = 1, 2, \cdots, 2N$), and the bottom surface dimensions of Layer-i match the top surface dimensions of Layer-(i+1) for $i \leq 2N$. The area of the top surface of the volume enclosed by Layer-i is given by $S_i = a_i b_i$.

The volume enclosed by each layer is calculated using the prismatoid volume equation, which is used for calculating the volume of variable-cross-section structures with two parallel end faces [42]. Specifically, the initial volume enclosed by Layer-i is given by:

$$V_{O,i} = \frac{h[S_i + S_{i+1} + (a_i + b_{i+1})(a_{i+1} + b_i)]}{6} \tag{16}$$

When the actuator deforms, the layer height h is replaced by the effective height $h_i(q_j)$, where $h_i(q_j)$ is determined by the bending angle q_j of the corresponding link. The effective height $h_i(q_j)$ is expressed as: $h_i(q_j) = d_i \sin q_j + h \cos q_j$.

Hence, the deformed volume $V_{D,i}$ enclosed by Layer-i is given by:

$$V_{D,i} = \frac{h_i[S_i + S_{i+1} + (a_i + b_{i+1})(a_{i+1} + b_i)]}{6} \tag{17}$$

The total volume change ΔV_j of Unit-j is the sum of the volume changes of adjacent layers. Based on the geometric composition of each unit (single-layer for the first and last units, double-layer for other units), ΔV_j can be expressed as follows:

$$\Delta V_j = \begin{cases} V_{D,1} - V_{O,1} & j = 1 \\ (V_{D,2j-2} - V_{O,2j-2}) + (V_{D,2j-1} - V_{O,2j-1}) & 2 \leq j \leq N \end{cases} \tag{18}$$

Based on the principle of virtual work, the work done by air pressure P on Unit-j (i.e., $P \cdot \Delta V_j$) is equal to the mechanical work done by torque M_j at the corresponding joint during angular deflection q_j. This relationship establishes the quantitative correlation between the volume change of the unit, the air pressure, the bending angle, and the output torque, which is expressed as:

$$M_j = P \cdot \frac{\Delta V_j}{\Delta q_j} \qquad j = 1, \ldots N \tag{19}$$

Each link satisfies torque balance condition, with the recursive form given as follows:

The first link ($j = 1$):

$$M_1 = L \cdot (F_z \sin(\beta_1) - F_x \cos(\beta_1)) \tag{20}$$

The Link-j ($2 \leq j \leq N$):

$$M_j - M_{j-1} = L \cdot (F_z \sin(\beta_j) - F_x \cos(\beta_j)) \tag{21}$$

where $L = 2h$ is the length of each link, and β_j denotes the cumulative bending angle of the Link-j which is the sum of the bending angles of all links from Link-N to Link-j, expressed as: $\beta_j = \sum_{k=j}^{N} q_k$ ($j = 1, 2, \ldots, N$).

The coordinate value of the tip end of the dual chamber actuator is obtained from forward kinematics:

$$X = \sum_{j=1}^{N} L \cos(\beta_j) \tag{22}$$

$$Z = \sum_{j=1}^{N} L \sin(\beta_j) \tag{23}$$

Hence, for a specified end position (X, Z) of the dual-chamber actuator, the corresponding tip force, F_x, F_z, and the bending angle of each link ($q_j, j = 1, 2, \ldots, N$) can be obtained by solving Eq. 20 to 23.

Note S4. Customized geometric parameters for the actuator

The top surface of the dual-chamber actuator is affixed to the proximal phalanx of the thumb via an MCP brace, as shown in Fig. S7(b). For aesthetic reasons, the cross-sectional area of the actuator should be approximately equal to or slightly smaller than the cross-sectional area of the subject's thumb. This design ensures adequate tip force while maintaining a streamlined appearance.

As shown in Fig. S11, the overall length of the dual-chamber actuator is given by:

$$H = Ns + 2t \tag{24}$$

where N represents the number of units, s is the height of a single layer, and t is the thickness of the upper and lower sealing plates shown in Fig. S11.

As described in the Experimental section, the dual-chamber actuator is initially manufactured and then compressed to its minimum length before being deployed on the thumb. The overall length H of the actuator is approximately twice its compressed length H'. The compressed length can be determined by measuring the distance between the MCP joint and CMC joint of the subject's thumb to ensure that the actuator covers an adequate range of motion.

The dual-chamber actuator is made of molding. To facilitate the demolding process, the initial crease angle α should be greater than 100°. Further, since the dual-chamber actuator is required to be compressed before the tempering process, the initial crease angle α should be less than 130° to guarantee the actuator can be compressed following the creasing.

The dimensions of the bottom surface of the actuator can be derived as follows:

$$a_{19} = a_1 + H'tan\theta \tag{25}$$

$$b_{19} = b_1 + H'tan\theta \tag{26}$$

Although a larger taper angle θ results in a greater tip force, it also increases the system volume. Therefore, a taper angle of 10° is selected. The major design parameters of the dual-chamber actuator are summarized in Table S5.

Note S5. Detailed process of control

The controller proposed in this paper implements an assist-as-needed (AAN) strategy to achieve the required thumb tip angle for specific elemental tasks while minimizing the assisting force. This approach enables stroke survivors to engage fully in training. The system employs Bayesian Optimization (BO) to meet its objectives. Additionally, it records the relative orientation θ_i of the two inertial measurement units (IMUs) and the assistive force F measured between the end of the dual-chamber actuator and the MCP brace.

Design of the Objective functions

When conducting elemental movements, the user performs the single movement several times. Therefore, the optimization must converge within a limited number of trials, allowing the user to fully engage in the optimized, high-intensity training. To achieve convergence within several cycles, two stages of optimization are implemented. When tip angles θ_i fall outside the acceptable range around the target, the controller prioritizes correcting the angular errors to quickly drive the actual angles into the target range. The objective function of the first stage is as follows:

$$J_1 = \omega_{r,1} \cdot R_{angle} \tag{27}$$

The objective function here is to achieve rapid convergence so that the user can complete the task without losing confidence. R_{angle} represents the preprocessed orientation θ_i, which aims to avoid over-penalizing angle deviations and enhance control robustness, as follows:

$$R_{angle} = \sum_{i=1}^{2} w_i \cdot r_i \tag{28}$$

where ω_i denotes the weight of the i-th angle, and r_i is the single-angle reward calculated as:

$$r_i = \begin{cases} 1.0 + \gamma(1.0 - \frac{|\theta_{act,i} - \theta_{tar,i}|}{\Delta}), & \text{if } L_i \leq \theta_{act,i} \leq U_i \\ max\left(0, 1.0 - \lambda \cdot d_i\right), & \text{if } \theta_{act,i} < L_i \text{ or } \theta_{act,i} > U_i \end{cases} \tag{29}$$

where:

- $\theta_{tar,i}$ is target angle for i-th degree-of-freedom.
- $\theta_{act,i}$ is actual angle for i-th degree-of-freedom.
- Δ is acceptable angle window to tolerate deviations.
- $L_i = \theta_{tar,i} - \Delta$ is the lower boundary of the target angle.
- $U_i = \theta_{tar,i} + \Delta$ is the upper boundary of the target angle.
- $d_i = \left|\theta_{\text{act},i} - \text{clip}\left(\theta_{\text{act},i}, L_i, U_i\right)\right|$ is the distance to the nearest window boundary.
- γ is the proximity bonus coefficient when actual angles are in the windows.
- λ is penalty coefficient when actual angles are outside the windows.

When θ_i fall into the target window Δ, the system switches the optimization focus to minimize assistive force and the objective function is:

$$J_2 = \omega_{r,2} \cdot R_{angle} - \omega_f \cdot P_{force} \tag{30}$$

Here, $\omega_{r,2}$ is angle rewards when the actual angles fall into the window, P_{force} is the normalized assistive force as follows:

$$P_{force} = 1 - \frac{1}{1 + \exp\left(-k \cdot (F - F_t)\right)} \tag{31}$$

where:

- $F = \sqrt{{F'_x}^2 + {F'_y}^2}$ is the magnitude of the baseline-corrected resultant interaction force.
- F'_x and F'_y represent the baseline-corrected interaction force, respectively.
- F_t is the threshold force used for normalization.

ω_f balances force minimization against angle maintenance, ensuring the system reduces assistance without compromising movement quality.

Bayesian optimization

Building on the previously discussed concepts, the optimization of assistance levels is refined using BO. This method is effective in optimizing objective functions in noisy environments and is known for its robustness and capability for global optimization. Central to this approach is the Gaussian Process (GP) model, which adjusts the pneumatic parameters during thumb rehabilitation training. This probabilistic optimization framework consists of the following components:

A composite kernel function is employed to establish a probabilistic mapping between pressure parameters and the objective function J:

$$k(\mathbf{x}, \mathbf{x}') = C \cdot exp(-\frac{1}{2}\sum_{d=1}^{2}\frac{(x_d - x'_d)^2}{l_d^2}) \tag{32}$$

where:

- $\mathbf{x} = (P_1, P_2)$ is the combination of two chambers' current pressure;
- $\mathbf{x}' = (P'_1, P'_2)$ is the combination of two chambers' historical pressure;

The BO algorithm executes the following iterative procedure:
Initial Sampling: Evaluate the objective function J at several initial pressure combinations.
Model Update: Construct the GP surrogate model using all historical observations.
Acquisition: Select the next evaluation point by maximizing the Expected Improvement (EI) criterion:

$$\mathbf{x}_{next} = \underset{\mathbf{x}\in\mathcal{X}}{\operatorname{argmax}} EI\left(x\right) \tag{33}$$

$$\mathbf{x}_{next} = \underset{\mathbf{x}\in\mathcal{X}}{\operatorname{argmax}} \left[(\mu_t(\mathbf{x}) - J^{+})\Phi(z) + \sigma_t(\mathbf{x})\phi(z)\right] \tag{34}$$

where:

- J^{+} represents the current best observation.
- $z = \frac{\mu_t(\mathbf{x}) - J^{+}}{\sigma(\mathbf{x})}$ standardizes the improvement.
- $\Phi(z)$ and $\phi(z)$ denote the cumulative distribution function and probability density function of the normal distribution.

This method does not rely on an explicit physical model. Instead, it uses real-time IMU and force-sensor data together with historical samples to build a Gaussian-process surrogate; Bayesian optimization then searches for pneumatic parameters that achieve the task with minimal assistance. In practice, this approach converges to a minimal effective assistance level while adapting to individual differences, in line with the Assist-as-Need principle.

Figure 3 illustrates the typical training control process of the system: First, user selects the task required on the application install on their smartphone, then the target angles for thumb movement for a specific task are set. When the amplitude of the electromyography (EMG) signal exceeds the trigger threshold, the optimization process starts. The system converges rapidly via Bayesian optimization, ensuring angles θ_i stably fall within the acceptable window while minimizing the assistive force F. After the optimal pressure parameters for movement assistance are finally determined, subsequent training is continuously conducted using these parameters, realizing a rehabilitation training mode of "AAN ".

References

[1] I. Jo, Y. Park, J. Lee, J. Bae, *Mechanism and Machine Theory* **2019**, *135* 176.

[2] M. B. Hong, S. J. Kim, Y. S. Ihn, G.-C. Jeong, K. Kim, *IEEE Transactions on Robotics* **2018**, *35*, 2 420.

[3] M. Bianchi, M. Cempini, R. Conti, E. Meli, A. Ridolfi, N. Vitiello, B. Allotta, *Mechatronics* **2018**, *51* 8.

[4] E. A. Susanto, R. K. Tong, N. S. Ho, *HKIE Transactions* **2015**, *22*, 2 78.

[5] A. Cisnal, J. Pérez-Turiel, J.-C. Fraile, D. Sierra, E. de la Fuente, *IEEE Access* **2021**, *9* 137809.

[6] V. Moreno-SanJuan, A. Cisnal, J.-C. Fraile, J. Pérez-Turiel, E. de-la Fuente, *Robotics and Autonomous Systems* **2021**, *143* 103828.

[7] M. Vangi, C. Brogi, A. Topini, N. Secciani, A. Ridolfi, *Machines* **2023**, *11*, 7 747.

[8] J. Vertongen, D. Kamper, In *Current Directions in Biomedical Engineering*, volume 6. De Gruyter, **2020** 20202003.

[9] H. Li, L. Cheng, N. Sun, R. Cao, *IEEE/ASME Transactions on Mechatronics* **2021**, *27*, 5 2699.

[10] D. Wang, Q. Meng, Q. Meng, X. Li, H. Yu, *IEEE Transactions on Neural Systems and Rehabilitation Engineering* **2018**, *26*, 12 2376.

[11] E. M. Refour, B. Sebastian, R. J. Chauhan, P. Ben-Tzvi, *Journal of mechanisms and robotics* **2019**, *11*, 6 060902.

[12] G. Li, L. Cheng, Z. Gao, X. Xia, J. Jiang, *IEEE Transactions on Robotics* **2022**, *38*, 6 3514.

[13] P. Agarwal, Y. Yun, J. Fox, K. Madden, A. D. Deshpande, *The International Journal of Robotics Research* **2017**, *36*, 3 355.

[14] F. Wang, C. L. Jones, M. Shastri, K. Qian, D. G. Kamper, N. Sarkar, *Advanced robotics* **2016**, *30*, 3 165.

[15] S. Ates, C. J. Haarman, A. H. Stienen, *Autonomous Robots* **2017**, *41*, 3 711.

[16] D. H. Kim, Y. Lee, H.-S. Park, *Soft robotics* **2022**, *9*, 4 734.

[17] P. Tran, S. Jeong, S. L. Wolf, J. P. Desai, *IEEE Robotics and Automation Letters* **2020**, *5*, 2 898.

[18] W. Chen, G. Li, N. Li, W. Wang, P. Yu, R. Wang, X. Xue, X. Zhao, L. Liu, *IEEE Transactions on Robotics* **2022**, *38*, 4 2194.

[19] K. B. Kim, H. Choi, B. Kim, B. B. Kang, S. Cheon, K.-J. Cho, *Soft Robotics* **2025**.

[20] B. Kim, H. Choi, K. Kim, S. Jeong, K.-J. Cho, *Soft Robotics* **2024**.

[21] M. Sui, Y. Ouyang, H. Jin, Z. Chai, C. Wei, J. Li, M. Xu, W. Li, L. Wang, S. Zhang, *Nature Machine Intelligence* **2023**, *5*, 10 1149.

[22] D. Serrano, D. Copaci, J. Arias, L. E. Moreno, D. Blanco, *IEEE Robotics and Automation Letters* **2023**, *8*, 9 5448.

[23] P. T. Phan, M. T. Thai, T. T. Hoang, N. H. Lovell, T. N. Do, *Ieee Access* **2020**, *8* 226637.

[24] S. Koizumi, T.-H. Chang, H. Nabae, G. Endo, K. Suzumori, M. Mita, K. Saitoh, K. Hatakeyama, S. Chida, Y. Shimada, In *2020 IEEE/SICE International Symposium on System Integration (SII)*. IEEE, **2020** 93–98.

[25] N. Takahashi, S. Furuya, H. Koike, *IEEE Transactions on Haptics* **2020**, *13*, 4 679.

[26] T. Zhang, K. Zheng, H. Tao, J. Liu, *Advanced Intelligent Systems* **2025**, *7*, 11 2500274.

[27] L. Ge, F. Chen, D. Wang, Y. Zhang, D. Han, T. Wang, G. Gu, *Soft robotics* **2020**, *7*, 5 583.

[28] L. Cappello, J. T. Meyer, K. C. Galloway, J. D. Peisner, R. Granberry, D. A. Wagner, S. Engelhardt, S. Paganoni, C. J. Walsh, *Journal of neuroengineering and rehabilitation* **2018**, *15*, 1 59.

[29] Y. Chen, X. Tan, D. Yan, Z. Zhang, Y. Gong, *IEEE journal of translational engineering in health and medicine* **2020**, *8* 1.

[30] J. Wang, Y. Fei, W. Pang, *Ieee/asme transactions on mechatronics* **2019**, *24*, 3 990.

[31] P. Polygerinos, Z. Wang, K. C. Galloway, R. J. Wood, C. J. Walsh, *Robotics and Autonomous Systems* **2015**, *73* 135.

[32] X. Shi, C. Yang, P. C. Lee, D. Xie, Z. Ye, Z. Li, R. K.-y. Tong, *Wearable Technologies* **2025**, *6* e4, edition: 2025/02/03.

[33] X.-Q. Shi, C.-H. E. Ti, H.-Y. Lu, C.-P. Hu, D.-S. Xie, K. Yuan, H.-L. Heung, T. W.-H. Leung, Z. Li, R. K.-Y. Tong, *Neurorehabilitation and Neural Repair* **2024**, *38*, 8 595.

[34] D. Xie, Y. Su, X. Shi, Z. Li, R. K.-y. Tong, In *2024 IEEE International Conference on Robotics and Automation (ICRA)*. IEEE, **2024** 2993–2999.

[35] Y. Wang, S. Kokubu, Z. Zhou, X. Guo, Y.-H. Hsueh, W. Yu, *IEEE Robotics and Automation Letters* **2021**, *6*, 4 8450.

[36] C. Chase-Markopoulou, A. Sepehri, T. K. Morimoto, M. T. Tolley, In *2025 IEEE 8th International Conference on Soft Robotics (RoboSoft)*. IEEE, **2025** 1–8.

[37] D. Hu, J. Zhang, Y. Yang, Q. Li, D. Li, J. Hong, In *2020 IEEE/ASME International Conference on Advanced Intelligent Mechatronics (AIM)*. IEEE, **2020** 1840–1847.

[38] J. Sun, M. Feng, D. Yang, Y. Wei, G. Gu, *Soft Robotics* **2026**, 21695172251400152.

[39] P. Wang, J. Zang, Z. Dong, Z. Xing, J. Zhao, *Sensors and Actuators A: Physical* **2025**, 116177.

[40] M. H. Namdar Ghalati, S. Akbari, H. Ghafarirad, M. Zareinejad, *Journal of Bionic Engineering* **2023**, *20*, 3 967.

[41] H.-J. Su, *Journal of Mechanisms and Robotics* **2009**, *1*, 21008.

[42] C. Alsina, R. B. Nelsen, *A mathematical space odyssey: solid geometry in the 21st Century*, volume 50, The Mathematical Association of America, **2015**.

[43] D. Drotman, M. Ishida, S. Jadhav, M. T. Tolley, *IEEE/ASME Transactions on Mechatronics* **2018**, *24*, 1 78.

[44] S. Liu, J. Liu, K. Zou, X. Wang, Z. Fang, J. Yi, Z. Wang, *Journal of Mechanisms and Robotics* **2022**, *14*, 6 060912.

[45] S. Liu, Z. Fang, J. Liu, K. Tang, J. Luo, J. Yi, X. Hu, Z. Wang, *Frontiers in Robotics and AI* **2021**, *8* 614623.

[46] S. Tang, K. Tang, S. Wu, Y. Xiao, S. Liu, J. Yi, Z. Wang, *Frontiers in Robotics and AI* **2023**, *10* 1210217.

[47] M. S. Xavier, C. D. Tawk, Y. K. Yong, A. J. Fleming, *Sensors and Actuators A: Physical* **2021**, *332* 113199.

[48] Y. X. Mak, A. Dijkshoorn, M. Abayazid, *Advanced Intelligent Systems* **2024**, 2300666.

[49] Z. Fang, Y. Wu, Y. Su, J. Yi, S. Liu, Z. Wang, *Scientific Reports* **2023**, *13*, 1 12116.

[50] Q. Tan, Y. Chen, M. Zhou, S. F. Tong, S. Liu, R. K.-Y. Tong, *Advanced Science* **2026**, *13*, 2 e14441.

[51] X. Yang, N. Zhang, X. Huang, R. Bian, M. Feng, X. Zhu, G. Gu, *IEEE Robotics and Automation Letters* **2024**.

[52] O. Shorthose, L. He, A. Albini, P. Maiolino, In *Annual Conference Towards Autonomous Robotic Systems*. Springer, **2021** 238–248.

[53] J. Yoon, J. Yang, D. Yun, *IEEE Robotics and Automation Letters* **2024**.

[54] W. Chen, C. Xiong, C. Liu, P. Li, Y. Chen, *Soft robotics* **2019**, *6*, 4 495.

[55] H. Ma, J. Zhou, *Acta Mechanica Solida Sinica* **2023**, *36*, 1 1.

[56] J. Guo, Z. Li, J.-H. Low, Q. Han, C.-Y. Chen, J. Liu, Z. Liu, C.-H. Yeow, *Soft Robotics* **2023**, *10*, 4 737.

[57] Y. Zhu, K. Chu, X. Chen, X. Wang, H. Su, *Sensors and Actuators A: Physical* **2022**, *338* 113492.

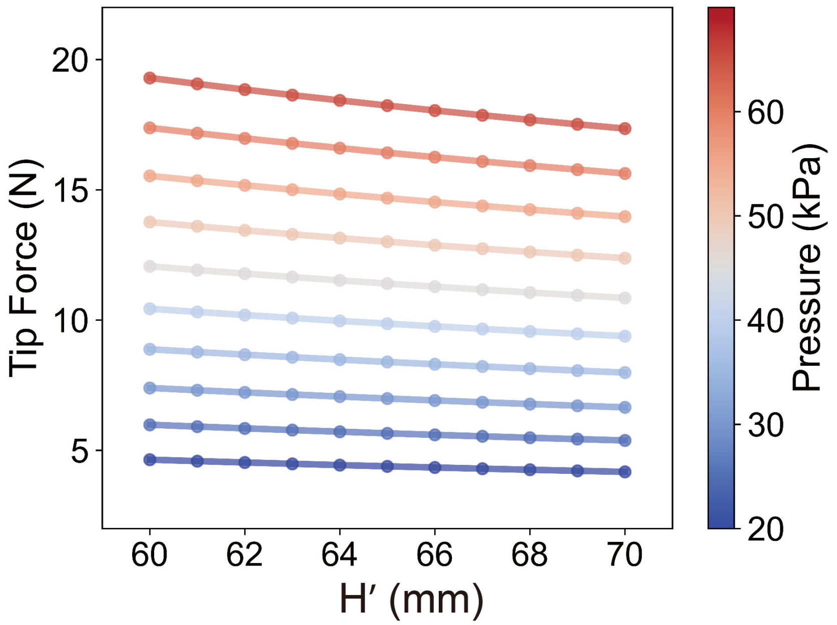

Figure S1: The modeled relationship between the tip force and the height of the actuator under different pressures.

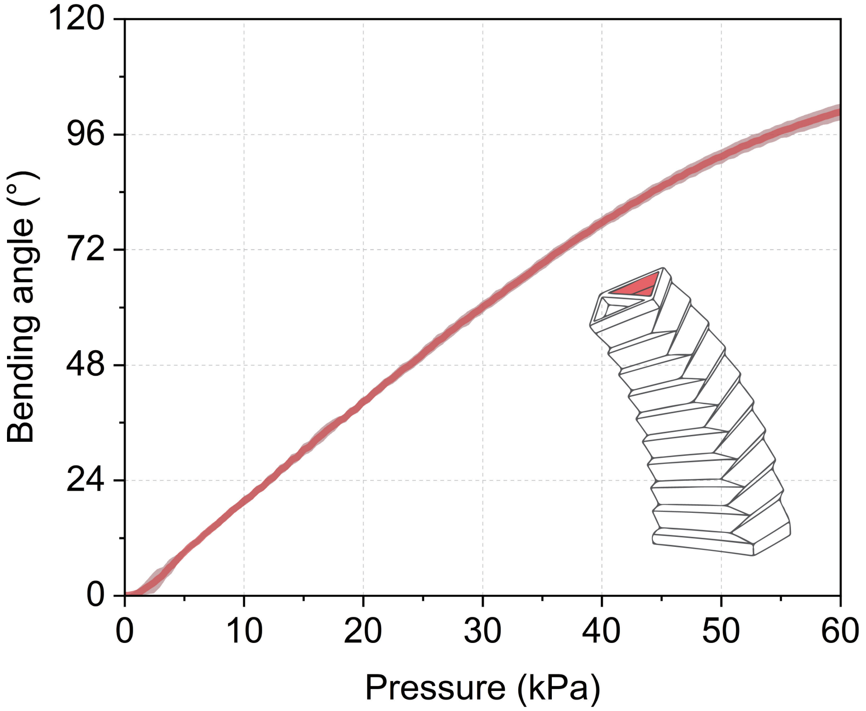

Figure S2: The tested relationship between pressure and bending angle when one chamber is inflated.

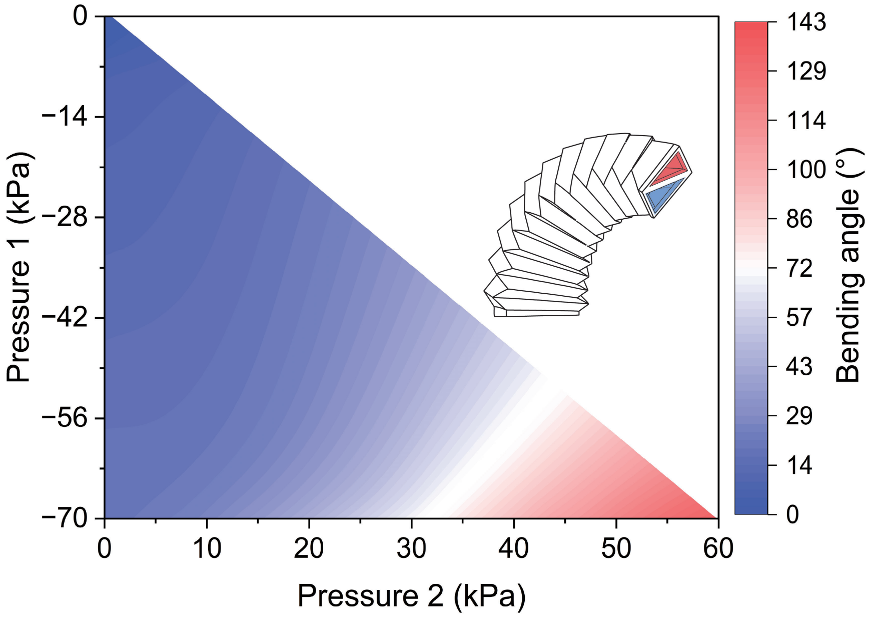

Figure S3: The tested relationship between pressure and bending angle when one chamber is inflated and the other chamber is vacuumed.

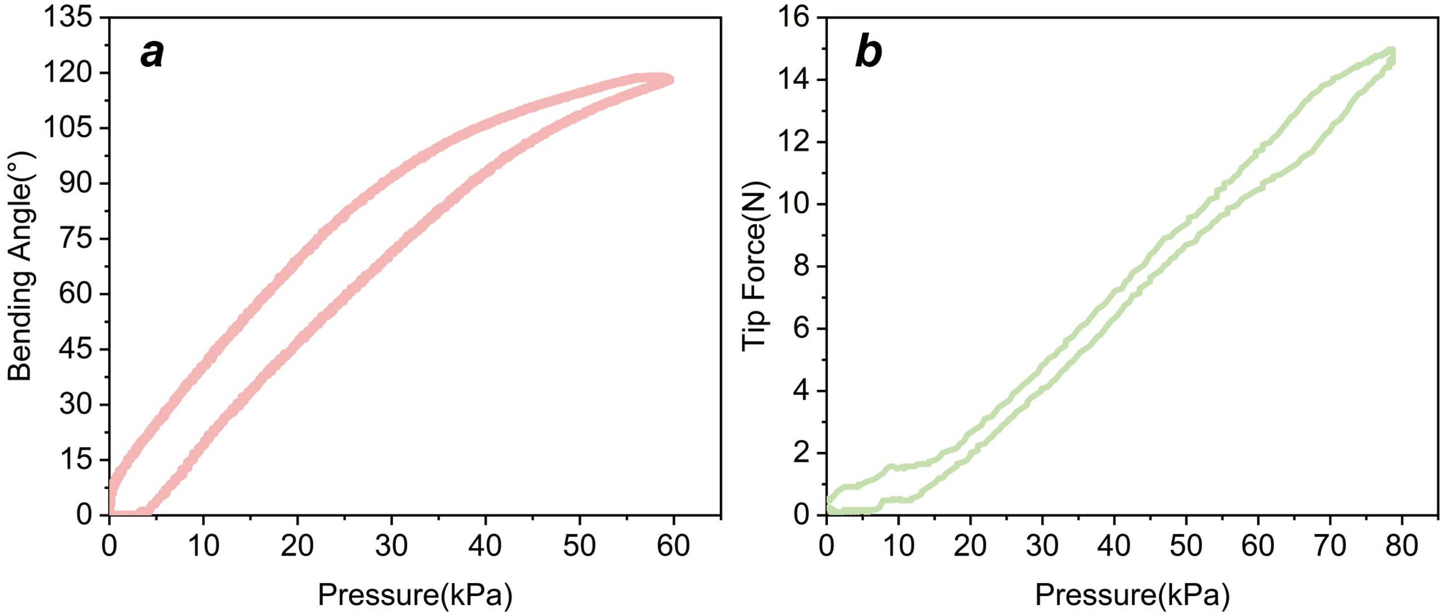

Figure S4: (a) The pressure-bending angle hysteresis loop. (b) The pressure-tip force hysteresis loop.

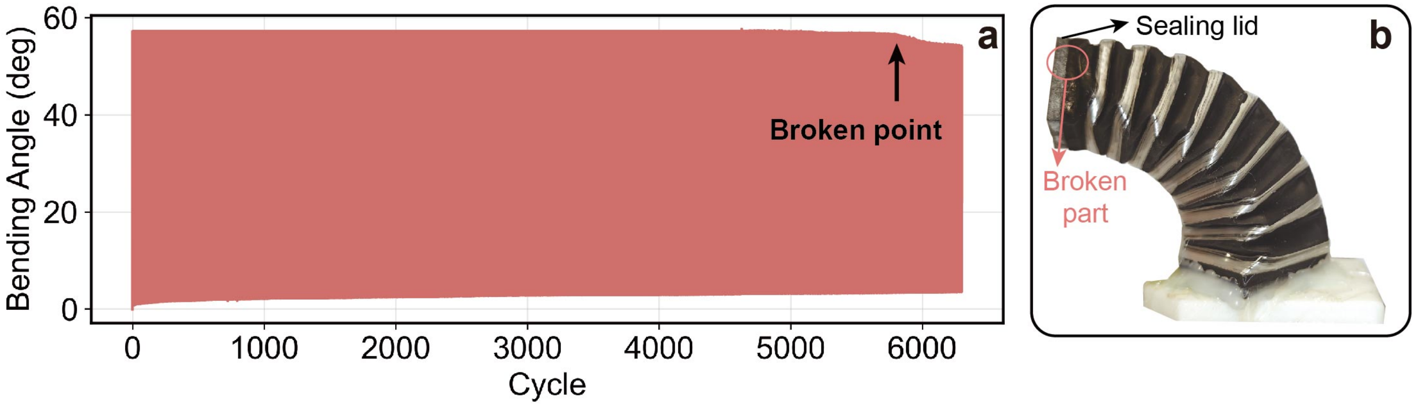

Figure S5: The lifetime of the actuator is tested using NOKOV motion capture system. (a) After 5850 cycles, the broken appears and the bending angle reduces by 4.2%. (b) The broken part is at the contact point between the top of the actuator and the sealing lid.

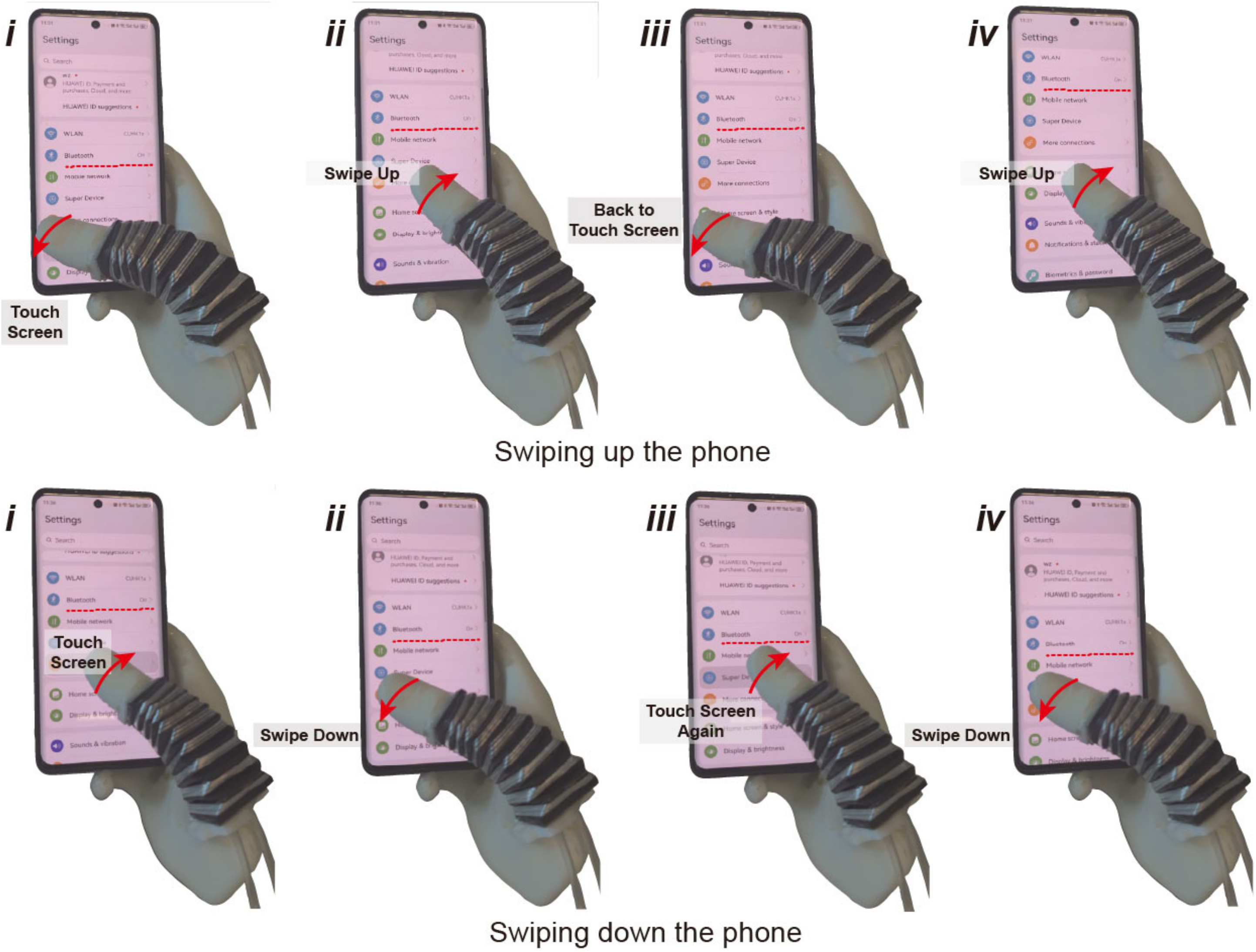

Figure S6: Swiping the phone using the dual-chamber actuator as the thumb of the model hand. (a) When swiping up the phone, ***i*** is the thumb bending to the lower left to touch the screen, ***ii*** is the thumb sliding to the upper right, and ***iii*** and ***iv*** repeat the actions of ***i*** and ***ii***.(b) When swiping down the phone, ***i*** is the thumb bending to the upper right to touch the screen, ***ii*** is the thumb sliding to the lower left, and ***iii*** and ***iv*** repeat the actions of ***i*** and ***ii***.

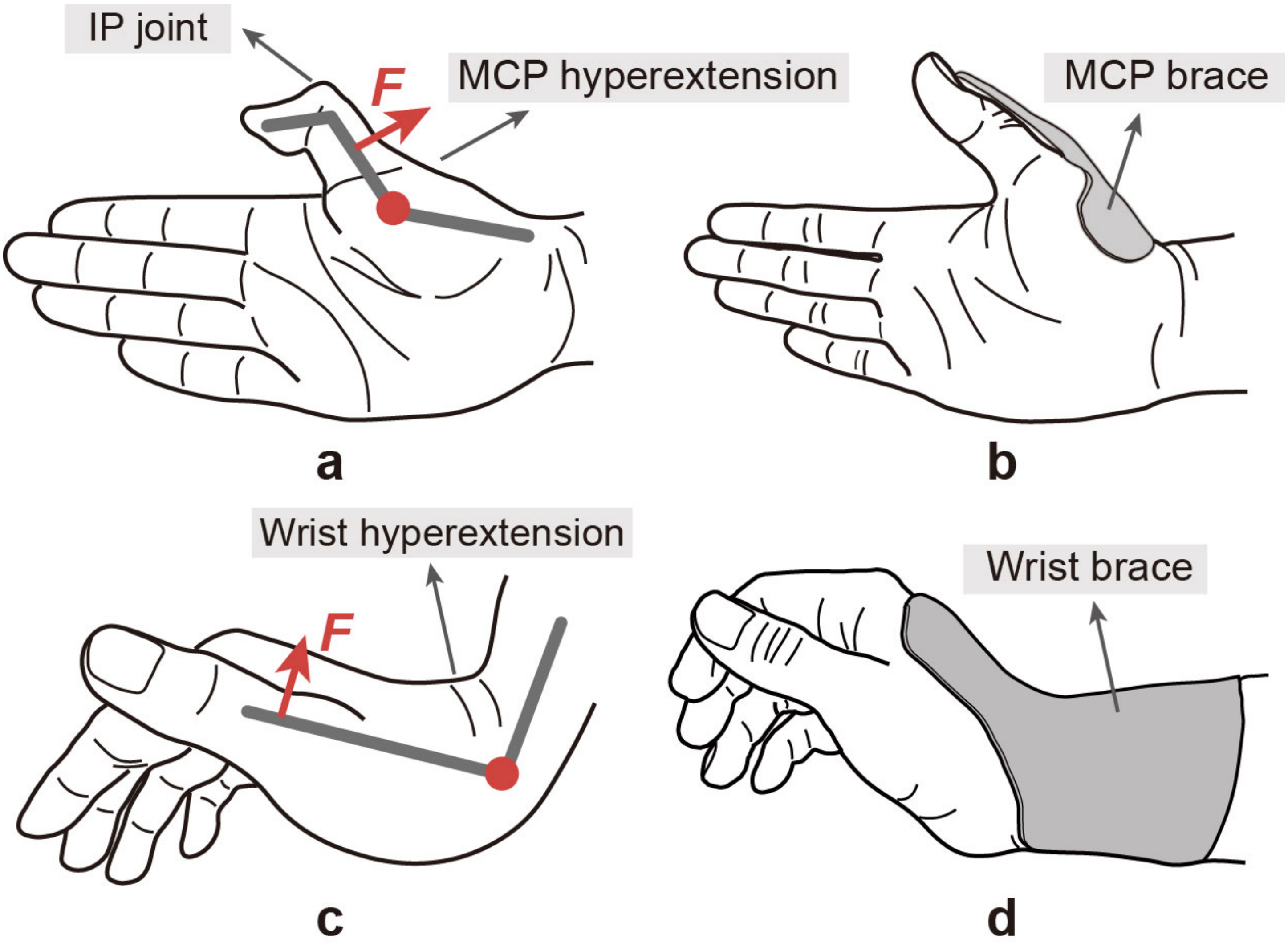

Figure S7: The actuator applies force to the proximal phalanx between the interphalangeal (IP) joint and the metacarpophalangeal (MCP) joint, which may lead to (a) MCP hyperextension. To address this, a MCP brace (b) is designed to fix the extension angle of the MCP joint and prevent hyperextension. The actuator's force may also cause (c) wrist hyperextension at certain angles. Therefore, a wrist brace (d) is designed to prevent hyperextension.

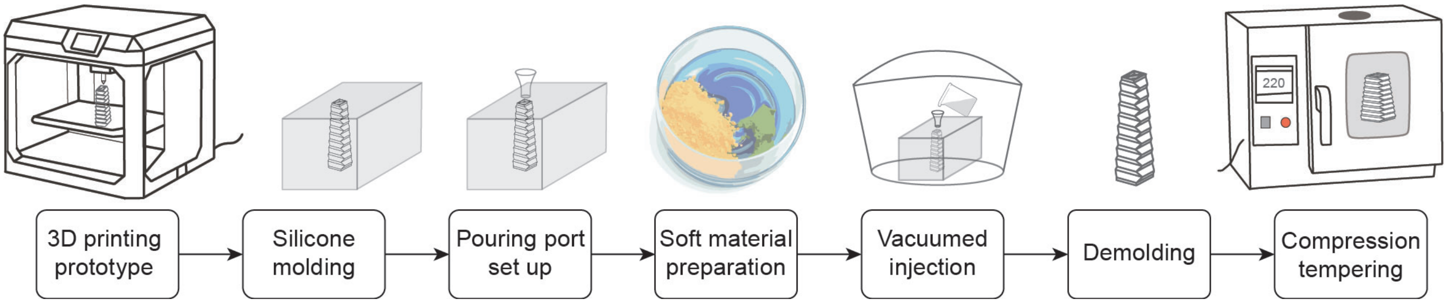

Figure S8: Fabrication method of the dual-chamber origami actuator.

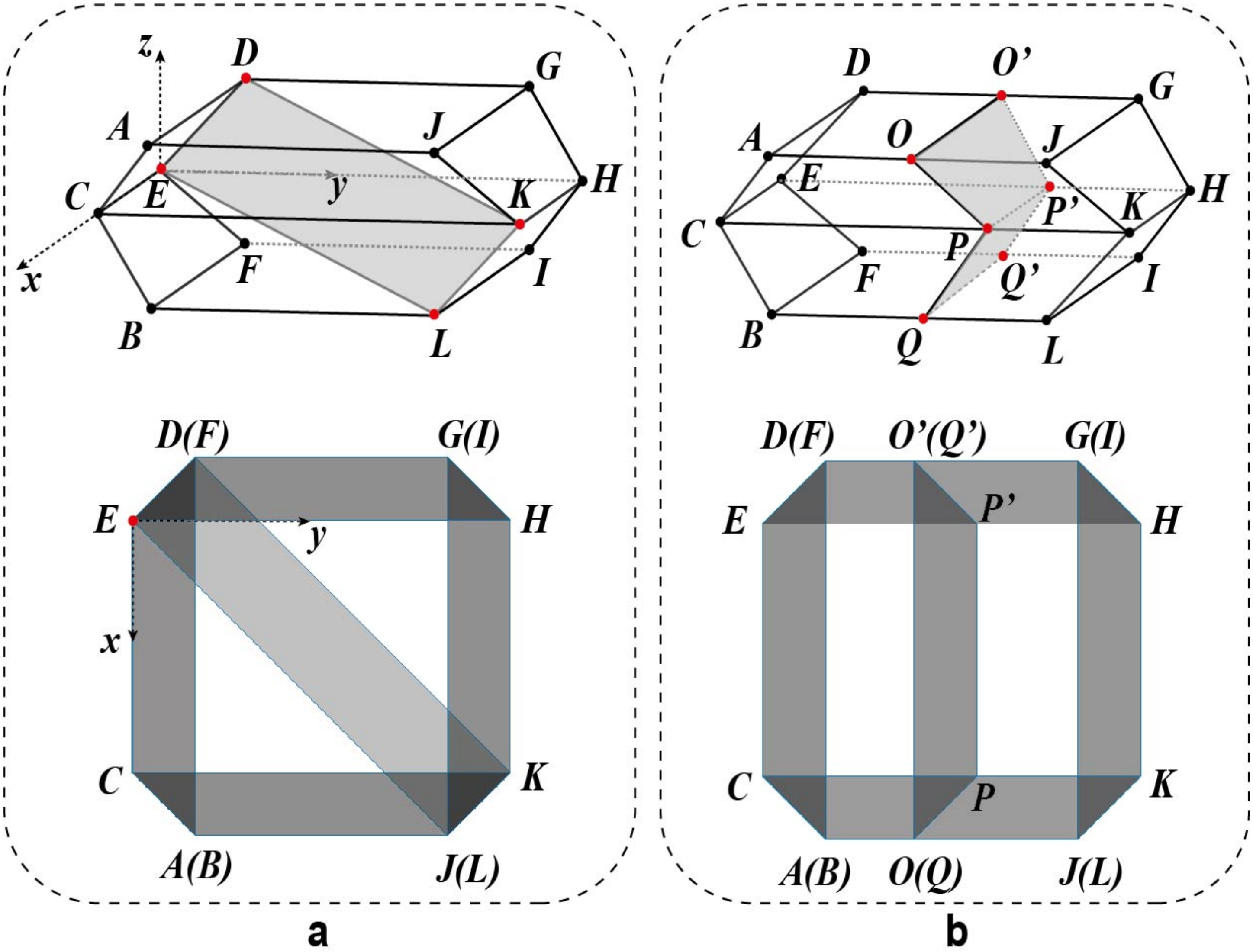

Figure S9: The comparison between (a) 'edge-edge-coupled' segmentation method and (b) 'edge-surface-coupled' segmentation method.

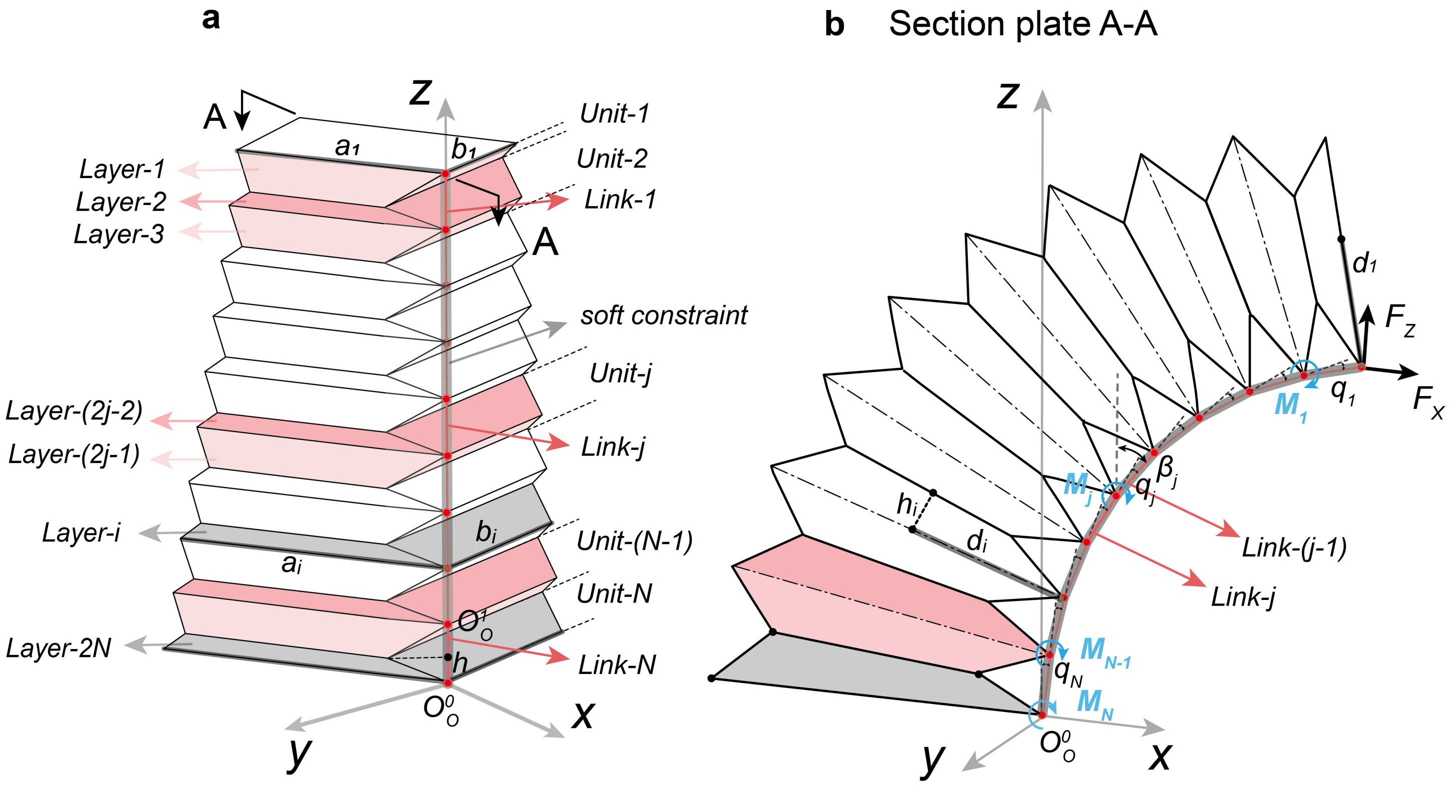

Figure S10: Modeling of the dual-chamber origami actuator. (a) The schematic diagram of the actuator with soft-constraint modeling. The section in (b) is formed from the diagonal planes of the actuator's upper and lower surfaces. (b) The detailed model of the structure. During actuator bending, each unit deforms coordinately. The total bending angle and tip force are achieved through the cumulative contribution of each unit.

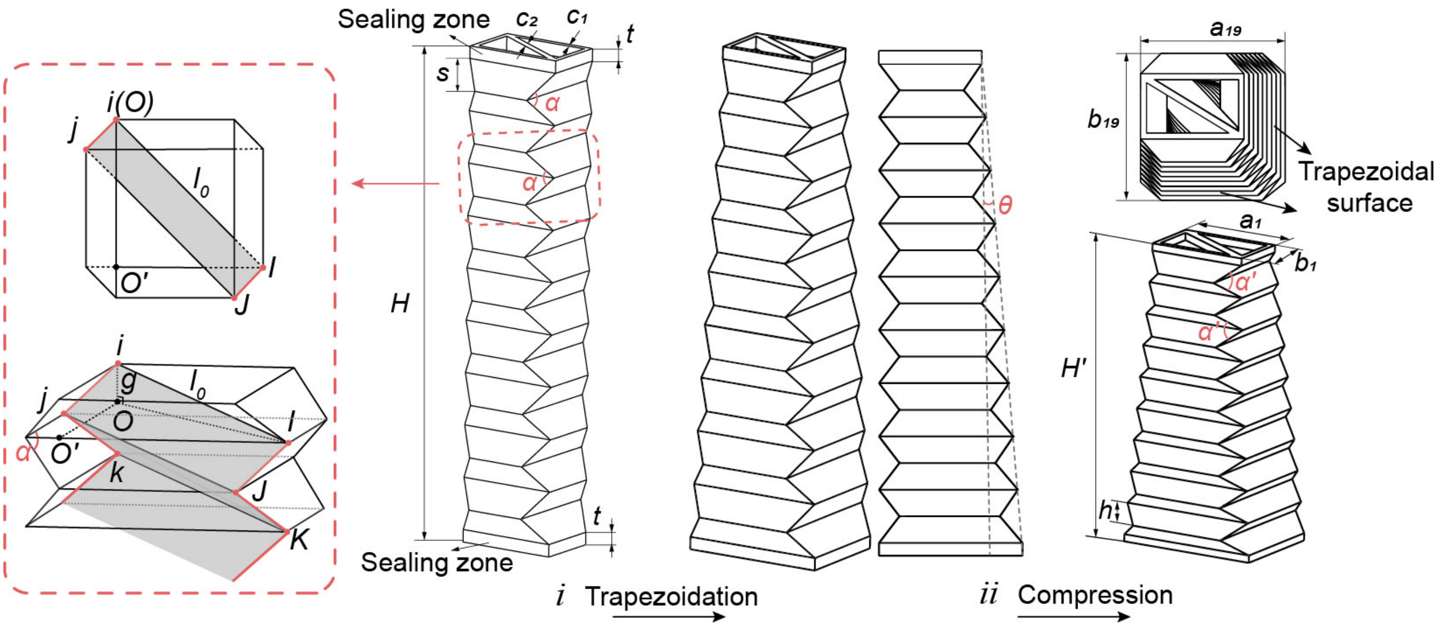

Figure S11: Dimensions of the dual-chamber actuator.

Table S1: The overview of the robotic devices that assist hand movement

Driven method	Name	Weight	T-DOFs	F-DOFs	TTF	FTF	Control	Tasks	Response Speed
Exoskeletons	Jo [1]	156 g	NA	fle, ext	NA	12 N	open-loop	NA	NA
	KULEX-Hand [2]	172 g	NA	fle, ext	NA	10 N	close-loop	grasp	NA
	HES [3]	NA	NA	fle, ext	NA	20 N	close-loop	grasp	NA
	Hand of hope [4]	NA	fle, ext	fle, ext	23 N	23 N	open-loop	open/close	NA
	RobHand [5,6]	600 g	fle, ext	fle, ext	23 N	23 N	close-loop	open/close	NA
	Vangi [7]	NA	fle, ext	fle, ext	NA	NA	close-loop	NA	NA
	Vertongen [8]	185g	fle, ext	fle, ext	NA	NA	open-loop	grasp	NA
	Li [9]	476 g	fle, ext	fle, ext	NA	12.3 N	close-loop	grasp	NA
	ReHand [10]	420 g	fle, ext	fle, ext	NA	NA	open-loop	grasp	NA
	Refour [11]	245 g	abd, add, fle, ext	fle, ext	20 N	20.9 N	close-loop	grasp	NA
	Li [12]	206 g	abd, add, fle, ext	NA	12 N	NA	close-loop	grasp	NA
	Maestro [13]	NA	abd, add, fle, ext	fle, ext	NA	NA	close-loop	grasp/pinch	NA
	ATX [14]	250g	abd, add, fle, ext	NA	∼25 N	NA	close-loop	NA	NA
	Agarwal [15]	4736 g	abd, add, fle, ext	NA	∼6 N	NA	close-loop	NA	NA
Motor-cable	Park [16]	NA	fle, ext	fle, ext	NA	NA	close-loop	grasp, pinch, swipe	NA
	FLEXotendon II [17]	297 g	fle, ext	fle, ext	5.03 N	NA	open-loop	grasp, pinch	5 s
	Chen [18]	<200 g	abd, add, fle, ext	fle, ext	10 N	NA	open-loop	grasp, pinch, circle	NA
	Exo-Glove Poly III [19]	408 g	add, fle, ext	fle, ext	∼6.9 N	NA	open-loop	grasp, pinch, close	NA
	Exo-Glove Shell [20]	NA	abd, add, fle, ext	fle, ext	4.08 N	NA	open-loop	grasp, pinch	8.62 s
Artificial muscle	Sui [21]	490 g	fle, ext, oppp	fle, ext	NA	14.3 N	close-loop	grasp, pinch, open/close	5.3 s
	Serrano [22]	2700 g	fle, ext, oppo	fle, ext	NA	NA	close-loop	grasp, pinch	4 s
	HFAM [23]	NA	fle, ext	fle, ext	NA	NA	open-loop	grasp	NA
	Koizumi [24]	64.7 g	fle, ext	fle, ext	NA	NA	open-loop	grasp	NA
	Takahashi [25]	NA	fle, ext	fle, ext	NA	8 N	open-loop	NA	NA
Bending Actuator	MFPMU glove [26]	NA	fle, ext	fle, ext	NA	2.34 N	open-loop	grasp, pinch	0.17 s
	Ge [27]	128 g	abd, fle	fle, ext	NA	38.5 N	close-loop	grasp	NA
	Cappello [28]	77 g	fle, ext	fle, ext	NA	NA	open-loop	grasp, pinch	NA
	Chen [29]	NA	fle	fle	NA	∼11.8N	open-loop	grasp, pinch	2 s
	Wang [30]	<300 g	fle	fle	NA	1.6 N	open-loop	grasp	NA
	Polygerinos [31]	285g	fle	fle	8 N	8 N	close-loop	grasp, pinch	2.2 s
	Shi [32,33]	150 g	fle, ext	fle, ext	NA	∼3.8 N	open-loop	grasp, pinch	NA
	Xie [34]	NA	abd, ext	ext	NA	NA	open-loop	NA	NA
	Wang [35]	NA	abd, add, fle	fle	NA	NA	open-loop	NA	NA
	Thumb Orthosis [36]	NA	abd, ext	NA	∼3.5 N	NA	open-loop	NA	NA
	Hu [37]	149 g	abd, add, fle, ext	fle, ext	NA	1.9 N	open-loop	grasp, pinch	NA
	Sun [38]	123.5 g	adb, add, fle, oppo	abd, fle	8.3 N	NA	open-loop	grasp, twist	0.7 s
	**This work**	**90 g**	**adb, add, fle, ext, oppo**	fle, ext	**18 N**	∼3.8 N	**close-loop AAN**	**grasp, pinch, open/close circle, twist, swipe**	1.1 s

Note: abd: abduction, add: adduction, fle: flexion, ext: extension, oppo: opposition, T-DOFs: Thumb DOFs, F-DOFs: Finger DOFs, TTF: Thumb Tip Force, FTF: Finger Tip Force, AAN: Assist-as-need

Table S2: The parameters used for FEM.

FEM parameters	Values
Hyperelastic material model	Yeoh model
Poisson's ratio	0.45
C_{10}	1.40 mm
C_{20}	$-1.32e^{-2}$
C_{30}	$9.52e^{-4}$
Material density	1.27 g/cm^3
Elements	C3D4H
Geometric nonlinearity	ON$NLGEOM = True$
Contact type	Self-contact
Boundary conditions	Fixed support and Pressure load

Table S3: The comparison among the proposed actuator and the existing works.

Actuator configuration	Reference	Actuator type	Chamber number	DOFs	Normalized bending angle ($^\circ/mm$)	Normalized tip force (N/mm^2)	Pressure (Positive + Negative −)
Actuator array	Drotman [43]	Bellow	3	3	∼1.000	N/A	+/−
	Liu [44]	Origami	24	6	∼0.396	∼0.002	+/−
	Liu [45]	Origami	2	2	0.375	∼0.006	+/−
	Tang [46]	Origami	4	3	∼0.304	0.015	+/−
Four-chamber	Xavier [47]	Bellow	4	3	∼1.640	0.003	+/−
Three-chamber	Mak [48]	Origami	3	3	∼0.980	∼0.005	+/−
Dual-chamber	Fang [49]	Bellow	3	2	1.353	∼0.012	+/−
	Tan [50]	Origami-bellow hybrid	2	2	1.778	0.002	+/−
	Yang [51]	Large-stretch based	2	2	∼0.875	∼0.014	+
	Shorthose [52]	Bellow	2	2	∼1.708	∼0.002	+/−
	Yoon [53]	Bellow	2	2	∼1.140	∼0.006	+/−
	Chen [54]	Bellow	2	1	∼1.170	N/A	+
	Ma [55]	Bellow	2	1	∼0.583	N/A	+
	Guo [56]	Kirigami	2	2	2.007	0.017	+/−
	Zhu [57]	Bellow	2	2	∼0.608	0.001	+
	This work	Origami	2	2	**2.200**	**0.027**	+/−

Note: Normalized bending angle = Bending angle/Length; Normalized tip force = Tip force/S_{ave}

Table S4: Demographic information of the subjects. FMA-UE: 0-66, ARAT: 0-57, MAS: 0-5

Subject	Gender	Age	Stroke type	Affected side	Stroke onset (years)	FMA-UE	ARAT	MAS	Disability level based on FMA-UE
S1	Male	55	Hemorrhagic	Left	6	24	22	0	Severe
S2	Female	63	Ischemic	Left	22	39	26	1	Moderate
S3	Female	44	Ischemic	Right	36	51	47	3	Mild

Table S5: Geometry parameters of the dual-chamber actuator prototype.

Geometry parameters	Symbols	Value
Initial actuator height	H	119.4 mm
Final actuator height	$H^{'}$	65.0 mm
Single layer height	s	6.3 mm
Initial crease Angle	α	105°
Final crease Angle	α'	20°
Unit number	N	9
External wall thickness	c_1	1.0 mm
Inner separator thickness	c_2	1.2 mm
Taper angle	θ	10°
Length of top surface	a_1	15.3 mm
Width of top surface	b_1	24.3 mm
Thickness of the sealing area	t	3.0 mm
Length of bottom surface	a_{19}	34.0 mm
Width of bottom surface	b_{19}	34.5 mm
